# Assessment of the microbial contamination in “Do It Yourself” (DIY) stores - a holistic approach to protect workers’ and consumers’ health

**DOI:** 10.3389/fpubh.2024.1483281

**Published:** 2024-10-17

**Authors:** Marta Dias, Bianca Gomes, Pedro Pena, Renata Cervantes, Sara Gonçalves, Elisabete Carolino, Magdalena Twarużek, Robert Kosicki, Iwona Ałtyn, Liliana Aranha Caetano, Susana Viegas, Carla Viegas

**Affiliations:** ^1^H&TRC – Health & Technology Research Center, ESTeSL – Escola Superior de Tecnologia e Saúde, Instituto Politécnico de Lisboa, Lisbon, Portugal; ^2^NOVA National School of Public Health, Public Health Research Centre, Comprehensive Health Research Center, CHRC, REAL, CCAL, NOVA University Lisbon, Lisbon, Portugal; ^3^CE3C—Center for Ecology, Evolution and Environmental Change, Faculdade de Ciências, Universidade de Lisboa, Lisbon, Portugal; ^4^Department of Physiology and Toxicology, Faculty of Biological Sciences, Kazimierz Wielki University, Bydgoszcz, Poland; ^5^Research Institute for Medicines (iMed.uLisboa), Faculty of Pharmacy, University of Lisbon, Lisbon, Portugal

**Keywords:** occupational exposure assessment, DIY stores, woodworkers, wood dust, fungi

## Abstract

**Introduction:**

In “Do-It-Yourself” (DIY) stores, workers from the wood department are considered woodworkers. Given the health risks associated with woodworking, particularly from fungi and their metabolites, this study aims to assess microbial contamination and health risks for both workers and customers.

**Methods:**

The study was developed in 13 DIY stores in Lisbon Metropolitan Area, Portugal. It employed a comprehensive sampling approach combining active (MAS-100, Andersen six-stage, Coriolis *μ*, and SKC Button Aerosol Sampler) and passive (electrostatic dust collectors, surface swabs, e-cloths, settled dust, filters from vacuumed dust, filtering respiratory protection devices, and mechanical protection gloves) methods to assess microbial contamination. A Lighthouse Handheld Particle Counter HH3016- IAQ was used to monitor the particulate matter size, temperature, and humidity.

**Results:**

The wood exhibition area presented the highest fungal load, while the payment area exhibited the highest bacterial load. MAS-100 detected the highest fungal load, and surface swabs had the highest bacterial load. *Penicillium* sp. was the most frequently observed fungal species, followed by *Aspergillus* sp. Mycotoxins, namely mycophenolic acid, griseofulvin, and aflatoxin G1, were detected in settled dust samples and one filter from the vacuum cleaner from the wood exhibition area. Cytotoxicity evaluation indicates the wood-cutting area has the highest cytotoxic potential. Correlation analysis highlights relationships between fungal contamination and particle size and biodiversity differences among sampling methods.

**Discussion:**

The comprehensive approach applied, integrating numerous sampling methods and laboratory assays, facilitated a thorough holistic analysis of this specific environment, enabling Occupational and Public Health Services to prioritize interventions for accurate exposure assessment and detailed risk management.

## 1 Introduction

The retail market for products used in home repair and improvement tasks, such as hardware, tools, building supplies, lawn, and garden products, is known as the DIY (Do-It-Yourself) market. These products are targeted at people who would choose to do DIY home renovation work rather than hiring a contractor (1). Despite the SARS-CoV-2 pandemic and the problems with the global supply chain, the major participants in the DIY and Hardware store industry grew significantly. This may be justified by the restrictions imposed by the pandemic which led to many individuals being confined to their homes, giving them time to work on home renovation projects. The DIY market is anticipated to expand by 3.52% a year (2), and it is divided into seven different segments, one of which is the Hardware and Building Materials segment, which comprises a diverse range of products including building materials such as wood (1). The wood sold in these stores can be cut by workers from the wood department, according to the measurements stipulated by the customer. Employees in retail stores devote a significant portion of their workday to receiving and unpacking merchandize, arranging items on hooks or shelves, and gathering merchandize for delivery to the showroom (3). In DIY stores, in addition to these duties, workers from the wood department also cut wood as part of their job description and are, therefore, considered woodworkers, making this a peculiar occupational setting due to a major source of contamination inside. However, there were no studies reporting microbial occupational exposure in DIY stores.

Exposure to wood dust is an occupational concern for millions of workers worldwide (4) since it is classified by the International Agency for Research on Cancer (IARC) as carcinogenic to humans (Group 1) (5). Wood dust is composed of fine particles of solid matter, and these solid particles are known as particulate matter (PM) (6). Particulate matter is a significant source and reservoir for microorganisms (7) acting as a carrier for the respiratory system facilitating the exposure by inhalation (8), being ingestion and dermal vias other exposure routes for PM (9), justifying the need for its evaluation in this study.

In addition to the PM, as published in other studies performed in sawmills (10–13), woodworkers are exposed to other potential health-harming agents, such as wood derivatives as well as microorganisms that grow on wood and their metabolites, which are influenced by environmental conditions like temperature and humidity. Reports of exposure to fungi, bacteria, and their metabolites—specifically, endotoxins—have already been made (11, 14–19). Although the health impacts of mycotoxin exposure through eating have been recognized (20, 21), not much research has been conducted regarding the health effects of mycotoxin exposure through inhalation, which is probably the main route of exposure in woodworking industries since the presence of PM facilitates the transport for the respiratory system (22). Therefore, the risk associated with the exposure remains unknown (23). Immunological mechanisms underlie most of the harmful effects caused by microorganisms associated with wood dust. According to previous studies, wood dust exposure can have a range of negative health impacts, including deep lung deposition that can lead to lung cancer, and reduced respiratory function, as well as nasal mucosa damage, irritation, and sino-nasal cancer (4, 24). The best-known are made by fungi, which can develop into secondary wood infections on wood products (chips, planks) that have not been stored under the correct conditions (23, 25). While the World Health Organization (WHO) has expressed concern about indoor biological agents in buildings and settled on a maximum value of 150 CFU/m^3^ for fungal exposure (26, 27), most countries have no regulations or proposed guidelines for acceptable concentrations of microorganisms in indoor environments, as known as occupational exposure limits (OEL) (27, 28). The lack of OEL happens due to the inexistence of information regarding agent-specific exposure-response correlations, considering the microbiological agents’ ability to reproduce in the presence of favorable conditions unlike other hazardous substances (29).

As in many other countries, the current Portuguese legislation lacks guidance on microbial occupational exposure for sawmills and related workplaces. The only guidance on microbial exposure adheres to microbial contamination in indoor environments (IAQ), ordinance n. 138-G/2021 (30), which differs for bacteria and fungi. Total bacteria should not exceed the outdoor by 350 colony-forming units and the fungal indoor concentrations should be lower than outdoor concentrations. If this criterion is not in compliance, a detailed assessment should identify fungal species and any visible growth in the environment (30).

The composition, concentration, and viability of the microbial contamination that is present in a specific setting are influenced by several sources, such as temperature and humidity, the presence of airborne organic particulate matter (27, 31) building occupancy, and the outdoor environment (27, 32). It is important to highlight that, in comparison to other examined indoor settings, DIY stores offer a distinct environment due to the volume of customers´ daily visits and products that come from a geographically diversified origin (33–35). Therefore, when focusing on the microbial contamination of DIY stores, it is crucial to consider the various exposure scenarios since these indoor environments are occupied by workers and customers. This study intends to characterize microbial contamination and the potential health risks for woodworkers and customers from DIY stores, aiming to reduce the adverse health effects while promoting good and safe working conditions.

## 2 Selection and characterization of the sampling sites and methods

### 2.1 “Do It Yourself” (DIY) stores’ selection and characterization

**Figure 1:**
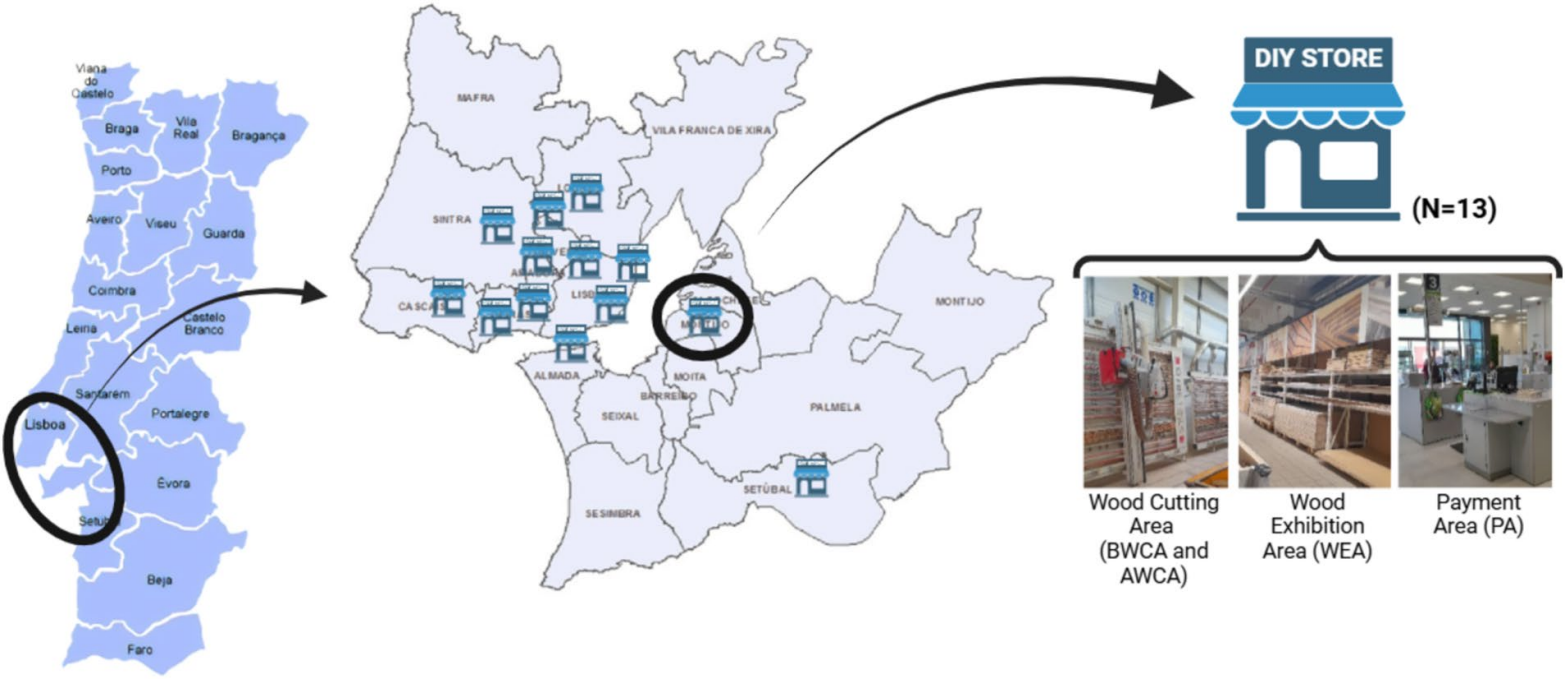
Geographical distribution of the DIY stores assessed and sampling sites (areas) identification.

A total of 13 DIY stores were selected from the Lisbon Metropolitan Area, in Portugal, between December 2022 and March 2023, and the sampling campaign was performed during an eight-hour work shift (08 h-17 h). Microbial occupational exposure was assessed in the wood-cutting area (WCA), before wood-cutting (BWCA), and after wood-cutting (AWCA), as well as in other areas of the DIY stores, such as the wood exhibition area (WEA) and the payment area (PA; Figure 1).

Outdoor samples (O) were also collected as a reference, and the PM (PM_0.3_, PM_0.5_, PM_1_, PM_2.5_, PM_5_, and PM_10_) were monitored, aiming to establish correlations between particle size and fungal contamination.

The main activities developed by DIY workers, in particular the ones that work in wood-cutting areas, included cutting different types of wood according to consumer requirements, removing the excess wood chips from the cutting machine, and removing the storage containers with wood chips collected during the cutting process.

**Figure 2:**
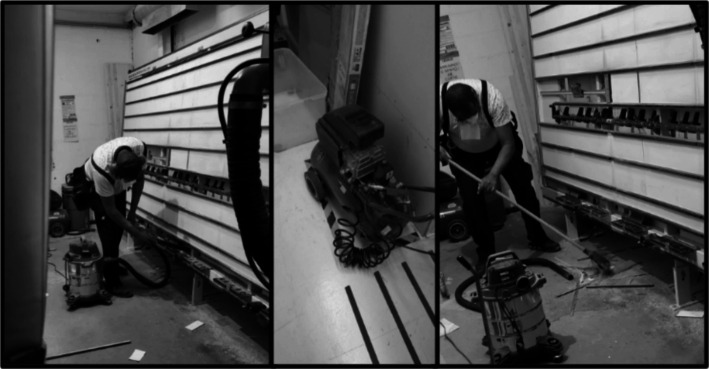
Cleaning procedures in the cutting machine.

In each DIY store, a walkthrough survey (Supplementary Table S1) was conducted to compile information about the daily number of workers and customers, the ventilation system adopted, the origin and types of wood used, the use of personal protective equipment (PPE), the cleaning and disinfecting procedures, as well as the waste management, in particular of the wood chips. This information (Supplementary Table S4), allowed the characterization of each store and the definition of the sampling sites. It was also used, along with the results, to develop a technical-scientific report provided to the company, recommending corrective measures based on the collected data. The wood-cutting area, the machine, and most of the wood dust on the floor are cleaned by the woodworkers, and once a day, the floor is cleaned and disinfected by the cleaning teams from each store. Throughout the day, the workers remove the wood chips, both from the cutting machine and from the floor, using a broom, a vacuum cleaner, or an air compressor (Figure 2). An analysis of the four sampling sites was developed considering the scientific and legal framework of both occupational exposure and indoor air quality, as well as the contextual information recovered with the walkthrough surveys, allowing us to identify the worst-case scenario regarding exposure to microbial agents in DIY stores.

### 2.2 Sampling campaign

#### 2.2.1 Particulate matter

To monitor the particulate matter, temperature, and humidity, the Lighthouse Handheld Particle Counter HH3016-IAQ was used at a flow rate of 2.83 L/min for 7 min (2 min for the equipment to stabilize and the other 5 min for the proper sampling). This monitoring was conducted at the level of the respiratory tract (whenever possible) in all sampling sites—WCA, WEA, PA, and O—in the WCA, samples were taken before and after cutting the wood.

#### 2.2.2 Microbial contamination

##### 2.2.2.1 Active sampling methods

**Figure 3:**
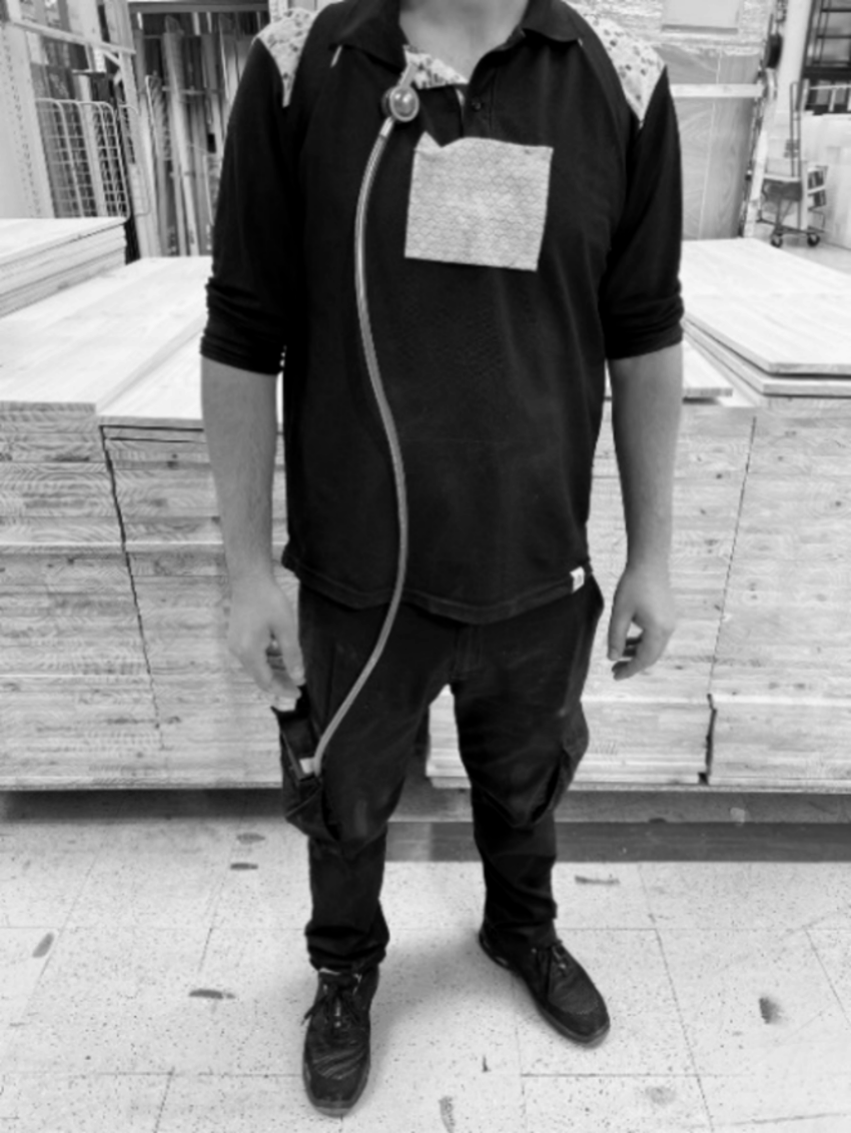
Personal sampling with a SKC Button Aerosol Sampler (active sampling) and an E-cloth (EDCP; passive sampling).

Samples from active sampling methods were collected in the same 4 sites mentioned above. The air sampling instruments used as stationary sampling in this study were a MAS-100 air sampler (Millipore, Billerica, United States) and, an Andersen six-stage cascade impactor (air impaction methods) with four different culture media to selectively collect fungi (malt extract agar (MEA) supplemented with chloramphenicol (0.05%), and dichloran-glycerol agar (DG18)) and for bacteria (tryptic soy agar (TSA) supplemented with nystatin (0.2%), and violet red bile agar (VRBA)). It is important to highlight that in the Andersen six-stage cascade impactor, the sampling with DG18 was doubled to allow incubation at two temperatures, 27°C, and 37°C to evaluate the pathogenic potential of fungi. For mycotoxins’ assessment, a Coriolis *μ* air sampler (Bertin Technologies, Montigny-le Bretonneux, France) was used. Besides stationary sampling, personal air sampling was also conducted on two workers, one from the WCA and the other from the WEA area using, the SKC Button Aerosol Sampler with a 0.8 μm 25 mm polycarbonate filter, connected to a SKC Universal air sampling pump (Figure 3). Sampling details are described in Supplementary Table S2.

##### 2.2.2.2 Passive sampling methods

**Figure 4:**
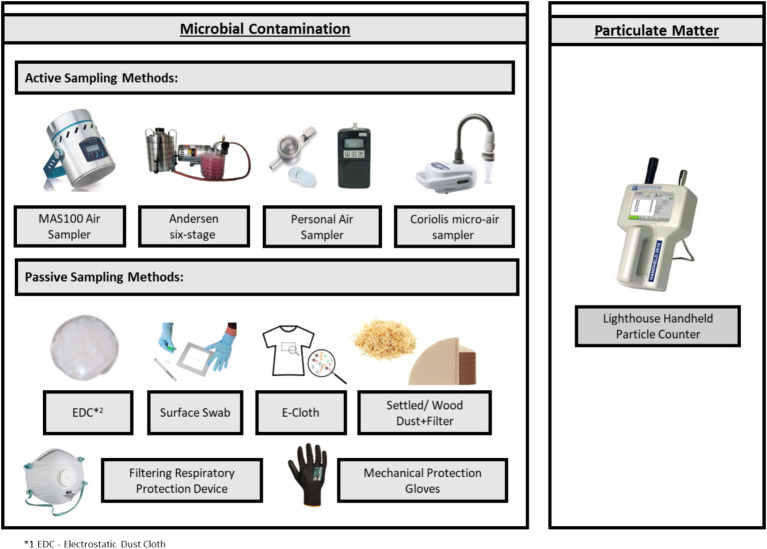
Sampling strategy (equipment and matrixes).

Samples from passive sampling methods were collected in the same four sites mentioned above—WCA, WEA, PA, and O. As passive sampling methods, Electrostatic dust collectors (EDC; Swiffer, Portugal), floor surface swabs (Frilabo, Portugal), e-cloths (EDCP; Swiffer, Portugal), vacuumed settled dust (SD; HOOVER Brave BV71_BV10 A2, United States), and filters from vacuumed dust (Continente, Portugal) were used. Furthermore, PPE, such as filtering respiratory protection devices (FRPD) and mechanical protection gloves (MPG), when used by the workers, were also recovered (Figure 4). All samples were maintained refrigerated (0–4°C) in sterilized bags prior to analysis (36) (Figure 4). Sampling details are described in Supplementary Table S2.

### 2.3 Sample extraction and characterization of viable microbiota

**Figure 5:**
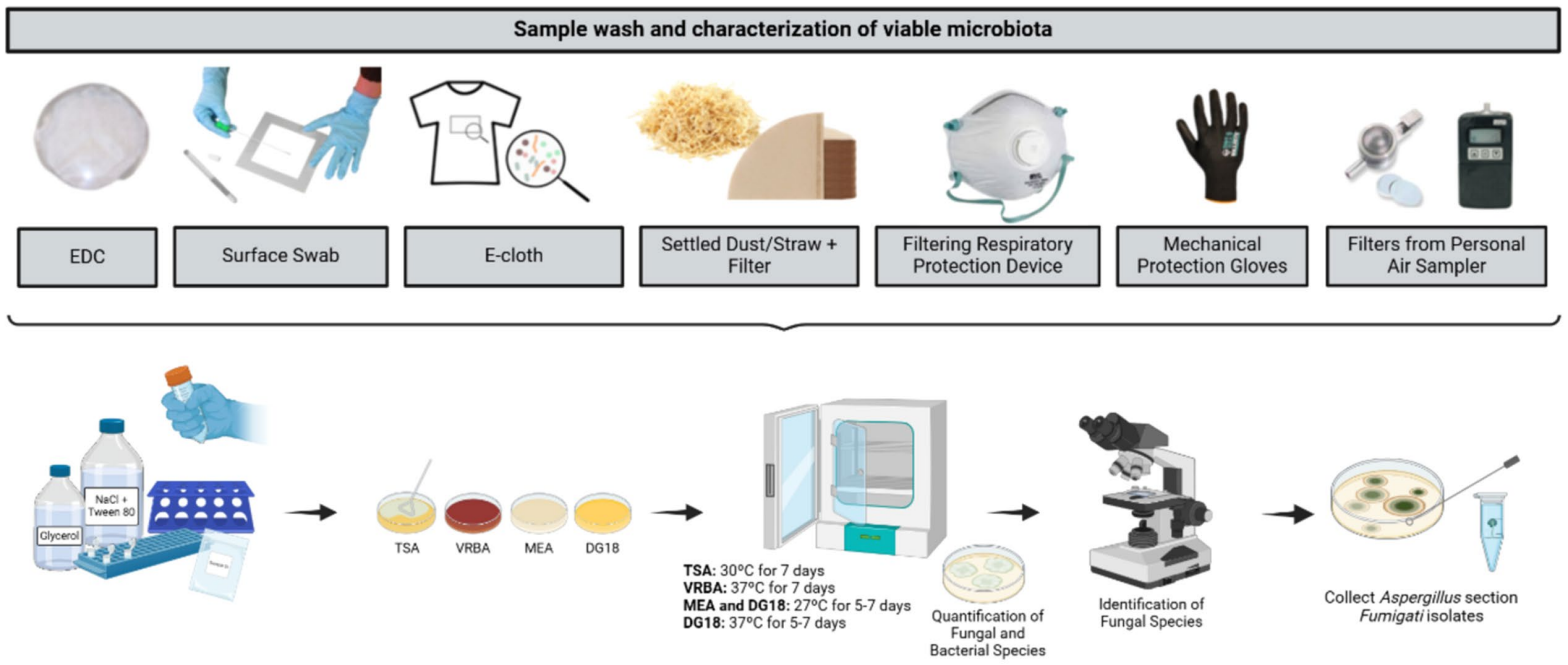
Laboratory work until microbial quantification and identification (Created with BioRender.com).

Passive samples were extracted according to a pre-established protocol (37) and the details are described in Supplementary Table S3. Similar to active sampling methods, MEA and DG18 were used for fungi (MEA and DG18, 27°C, 5–7 days; and an additional DG18 at 37°C, 5–7 days was used to evaluate the pathogenic potential), TSA and VRBA were used for bacteria, mesophilic (TSA, 30°C, 7 days), and Gram-negative (VRBA, 37°C, 7 days). The screening of azole-resistant fungi, was also developed according to a pre-established protocol (37) adapted from EUCAST guidelines (38, 39), all passive samples were seeded on sabouraud dextrose agar (SDA) supplemented with 4 mg/L itraconazole (ITZ), 2 mg/L voriconazole (VCZ), or 0.5 mg/L posaconazole (PSZ), and incubated for 48 h at 27°C. Microbiota quantification was determined as colony-forming units (CFU) and CFU concentration (CFU/m^3^/m^2^/m^2^ × day^1^/g^1^) after plate incubation. Morphological identification of fungal species was carried out by notating macro-and microscopic characteristics (40) by an environmental and occupational mycologist (Figure 5).

The number of samples/measures from each sampling method performed in DIY stores, as well as FRPD and MPG recovered, are summarized in Supplementary Table S4.

### 2.4 Mycotoxins analysis

A total of 237 samples were screened for the presence of mycotoxins (49 air samples collected by the Coriolis air sampler, 39 settled dust samples collected via vacuuming, 48 filters used on the vacuum cleaner, 34 EDC, 38 EDCP, 24 MPG, and 5 FRPD). The detection of mycotoxins was performed using a high-performance liquid chromatograph (HPLC) Nexera (Shimadzu, Tokyo, Japan), equipped with a mass spectrometry detector (5,500 Qtrap; Sciex, Foster City, CA, United States). The mycotoxins analyzed, as well as their Limits of Detection (LOD) are provided in Supplementary Tables S3, S5.

### 2.5 Cytotoxicity evaluation

A cytotoxicity evaluation was developed to identify the toxicity of the co-exposure to all the environmental contaminants present in this occupational setting. Coriolis air, settled dust, filters used on the vacuum cleaner, EDC, e-cloths, MPG, and FRPD samples were prepared by two-fold serial dilutions and used to evaluate the metabolic activity of human lung epithelial (A549) and swine kidney (SK) cells using the 3-(4,5-dimethylthiazol-2-yl)-2,5-diphenyltetrazolium bromide (MTT) assay based on a pre-established protocol (37) and the details are also described as Supplementary Methods S1.

### 2.6 Statistical analysis

The data was analyzed using the R-Cran software, version 4.3.3 for Windows. The results were considered significant at a significance level of 5%. To characterize the sample, frequency analysis was used for qualitative data and means and standard deviations for quantitative data, with graphical representations appropriate to the nature of the data. To test the normality of the data, the Shapiro–Wilk test was used. Regarding particulate matter, Principal Component Analysis (PCA) was carried out to reduce information. To evaluate the quality of the PCA, the Kaiser-Meyer-Olkin statistic (KMO = 0.636, revealing a reasonable quality) and the Bartlett test for sphericity were used [having rejected the hypothesis that the correlation matrix was the identity matrix (*p* < 0.05)]. To study the relationship between fungal contamination, bacterial contamination, azole resistance screening, particulate matter, and environmental conditions, Spearman’s correlation coefficient was used, since the assumption of normality was not verified. To assess species diversity, Simpson and Shannon indices, given by $\mathrm{Shannon}\;\mathrm{Index}\;\left(H\right)=-\overset{s}{\underset{i=1}{\sum }}p_{i}\ln\left(p_{i}\right)$ and $\mathrm{Simpson}\;\mathrm{Index}\;\left(D\right)=\frac{1}{\sum_{i=1}^{s}p_{i}^{2}}$ , where *p_i_* is the proportion (n_i_/n) of individuals of one particular species found (n_i_) divided by the total number of individuals found (n).

## 3 Results

### 3.1 Particulate matter measurements

The particulate matter levels were higher in the wood-cutting area after cutting (AWC) for all particle diameters, followed by the payment area (P) on three-particle diameters (Table 1).

**Table 1:** Descriptive statistics of all PM sizes per monitored site.

Monitoring site		μgr/m^3^			
		Min	Max	x̄	σ
WCA	0,3 μ	0.64	3.69	2.03	0.92
	0,5 μ	0.87	6.33	2.50	1.34
	1,0 μ	3.83	67.48	18.39	16.15
	2,5 μ	21.96	463.55	117.89	111.28
	5,0 μ	78.54	1639.3	446.30	393.64
	10,0 μ	97.01	1681.53	780.28	483.50
WEA	0,3 μ	0.46	3.62	1.83	0.92
	0,5 μ	0.63	3.52	1.74	0.89
	1,0 μ	3.88	20.92	8.57	5.43
	2,5 μ	14.18	89	34.69	22.05
	5,0 μ	25.66	180.06	78.41	49.82
	10,0 μ	22.85	142.38	62.64	44.04
PA	0,3 μ	0.32	4.57	1.75	1.11
	0,5 μ	0.58	3.33	1.66	0.72
	1,0 μ	1.33	13.46	7.16	3.78
	2,5 μ	7.06	62.32	30.96	17.90
	5,0 μ	21.79	188.42	75.98	46.28
	10,0 μ	16.97	142.91	56.60	32.65
O	0,3 μ	0.35	7.96	2.88	2.54
	0,5 μ	0.5	7.14	2.53	1.83
	1,0 μ	1.41	23.01	7.77	6.30
	2,5 μ	5.21	70.05	24.37	18.33
	5,0 μ	5.71	65.33	33.35	18.72
	10,0 μ	3.9	40.75	21.08	10.10

**Figure 6:**
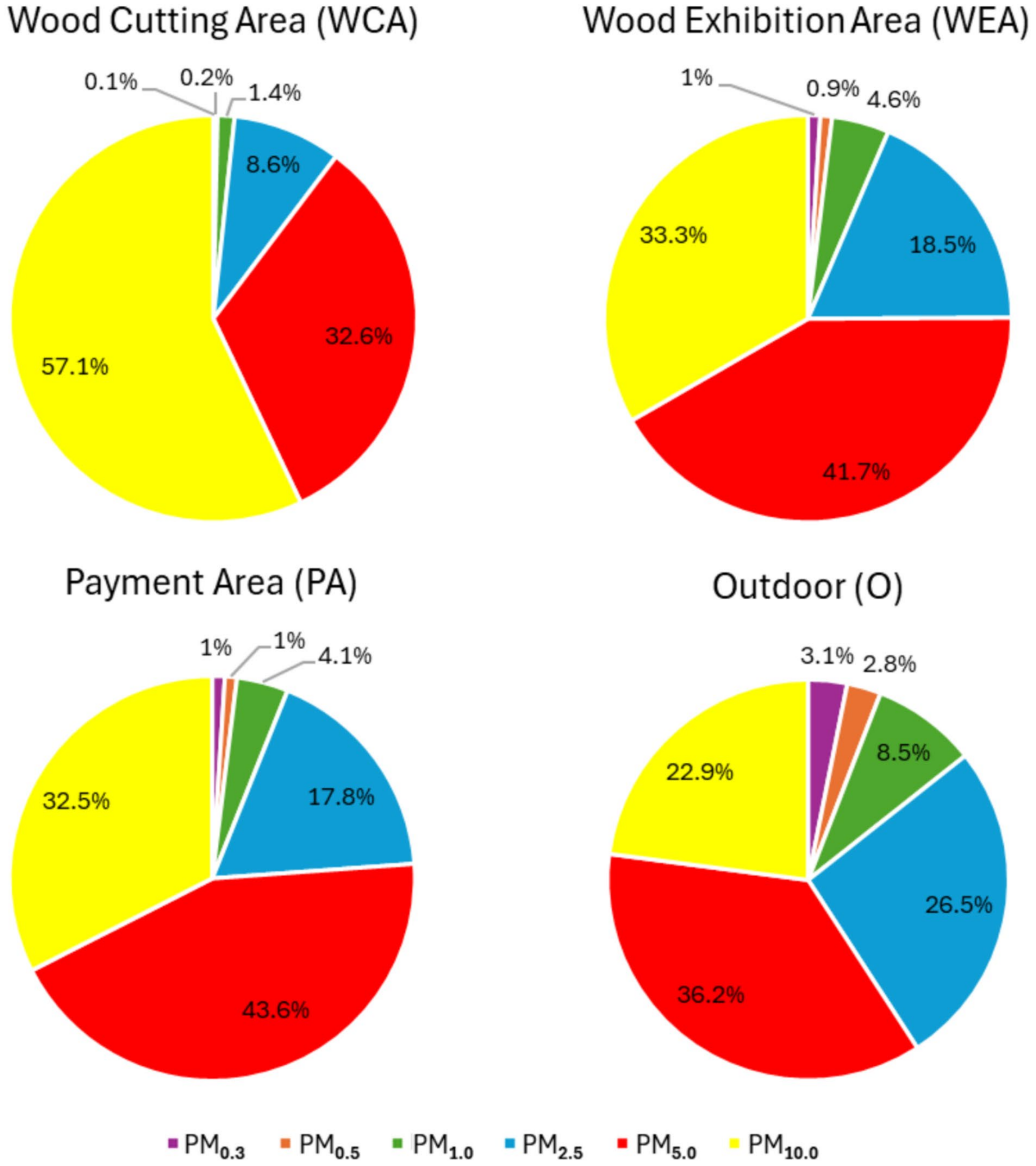
Prevalence of each particle size in each site.

Regarding the I/O ratio, for PM_2.5_, PM_5_, and PM_10_, all sampling sites had an I/O ratio > 1 indicating that increased concentrations indoors are caused by emission sources (e.g., activities developed) that are present indoors. Regarding the distribution of each particle diameter, PM_5_ presented the highest prevalence in most sampling sites (WEA—41.7%; PA—43.6%), followed by PM_10_ (WEA—33.3%; PA—32.5%). In the wood-cutting area, PM_10_ presented the highest prevalence (57.1%) followed by PM_5_ (32.6%). The outdoors presented the highest prevalence of inhalable particle sizes PM_5_ (36.2%), PM_2.5_ (26.5%), and PM_10_ (22.9%; Figure 6).

### 3.2 Definition of the worst-case scenario regarding microbial contamination within DIY stores

Considering the quantitative comparison (indoor/outdoor) of the fungal load, two stores showed higher indoor load than outdoor load at all sampling sites on MEA, whereas on DG18 five stores showed higher indoor contamination than outdoor contamination at all sampling sites.

Concerning the occupational exposure assessment and considering the threshold suggested by the European Agency for Safety and Health at Work (EU-OSHA) (29) for fungi in non-industrial workplaces (1.0 × 10^1^–1.0 × 10^4^ CFU/m^3^), all the sampling sites from all the stores are within the limit.

**Figure 7:**
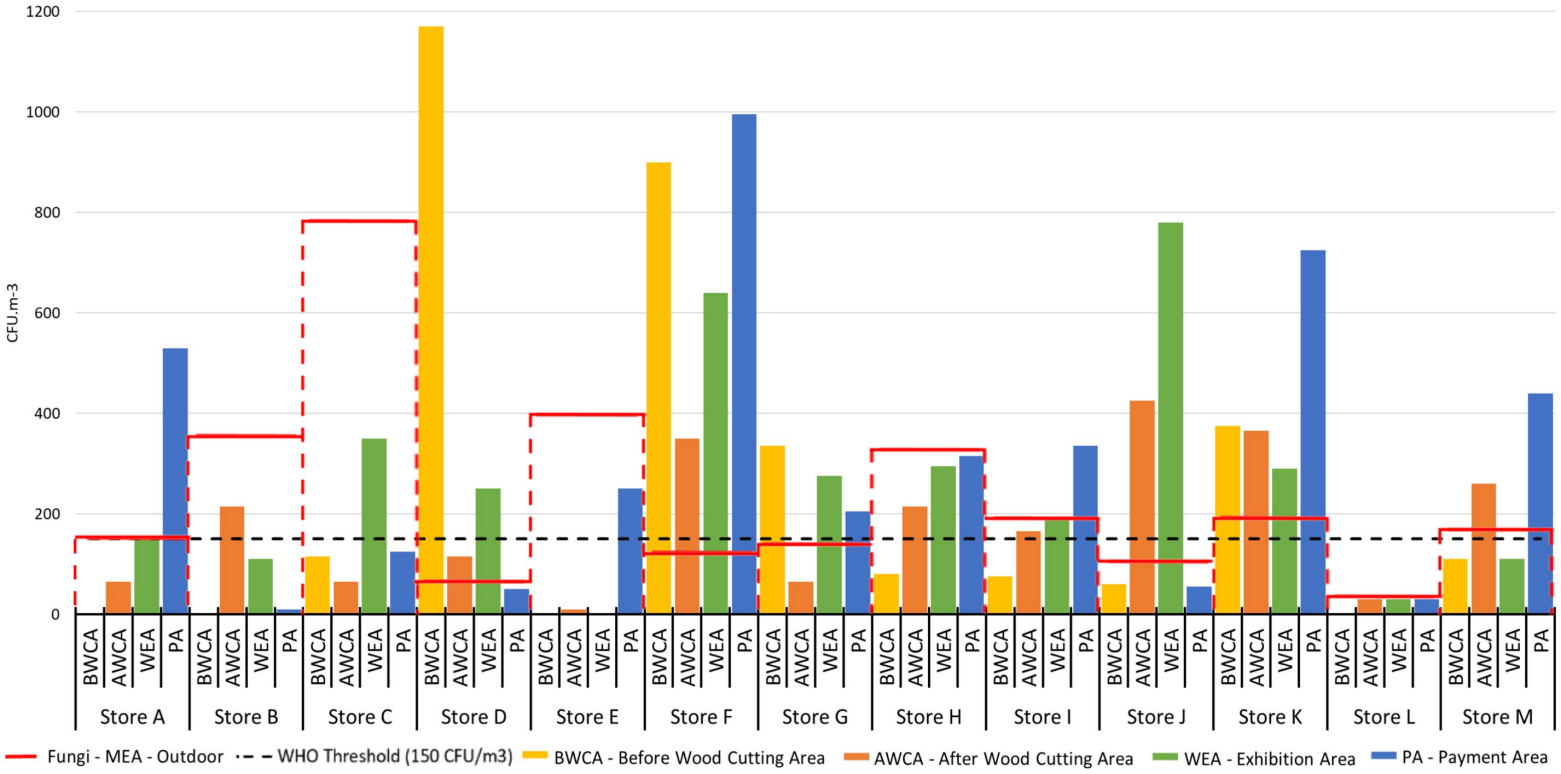
Fungal contamination per sampling site in each store on MEA (the red lines correspond to the concentration outdoor per store, the black non-continuous line corresponds to the WHO threshold).

Regarding the customers ‘exposure and applying the limit suggested by the WHO (150 CFU/m^3^), each sampling site was not compliant in several stores (Table 2; Figure 7; Supplementary Figure S1).

**Table 2:** Non-compliant stores considering the WHO (150 CFU/m^3^) limit.

	Sampling site	Number of non-compliant stores
MEA	BWCA	4 out of 13 (30.8%)
	AWCA	7 out of 13 (53.9%)
	WEA	8 out of 13 (61.5%)
	PA	8 out of 13 (61.5%)
DG18	BWCA	5 out of 13 (38.5%)
	AWCA	10 out of 13 (76.9%)
	WEA	10 out of 13 (76.9%)
	PA	9 out of 13 (69.2%)

Considering the Portuguese Indoor Air Quality (IAQ) legal framework (Ordinance n. 138-G/2021, de 1 de julho) (30), the ratio I/O complied in 5 out of 13 stores (38.5%) on MEA. However, it was possible to identify in one of these, one toxigenic species with a quantitative cut-off above the legal frame. On DG18, the ratio I/O did not comply in any store, and it was also possible to identify, in 6 out of the 13 stores (46.1%), three toxigenic species with a quantitative cut-off above the legal frame (Figure 7; Supplementary Figure S1).

Considering the quantitative comparison (indoor/outdoor) of the bacterial load, four stores (30.8%) showed higher indoor contamination than outdoor contamination at all sampling sites on TSA, whereas, on VRBA, no store showed higher indoor contamination than outdoor contamination at all sampling sites.

Concerning the occupational exposure assessment and considering the thresholds suggested by EU-OSHA for TSA in non-industrial workplaces (1.0 × 10^3^–7.0 × 10^3^ CFU/m^3^) and for VRBA for manufacturing and industrial premises (1.0 × 10^3^–2.0 × 10^4^ CFU/m^3^), all sampling sites from all stores are within the limit.

**Figure 8:**
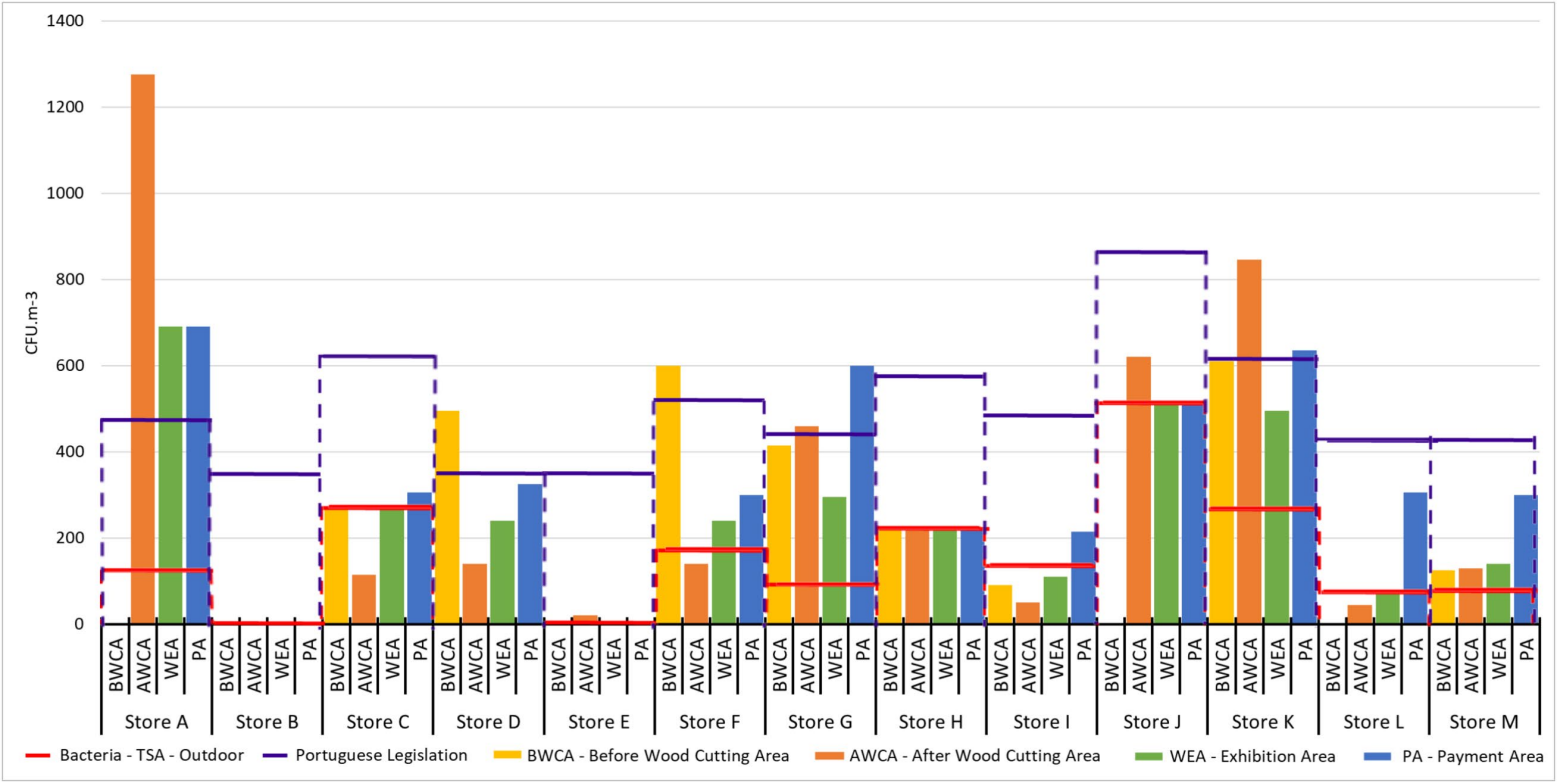
Bacterial contamination per sampling site in each store on TSA [the red lines correspond to the concentration outdoor per store, the purple lines correspond to the Portuguese Legislation (concentration outside, plus 350 CFU/m3)].

Regarding the customers’ exposure and considering the cut-off from the Portuguese IAQ legal framework (concentration of total bacteria inside lower than the concentration outside, plus 350 CFU/m^3^), 4 out of the 13 stores (30.8%) showed at least one sampling site with a quantitative cut-off above the legal frame. on TSA, and in 2 out of 13 stores (15.4%) on VRBA (Figure 8; Supplementary Figure S2).

Based on the results presented above, it was possible to identify that the worst-case scenario within DIY stores, is the wood exhibition area (WEA) for fungal load and the payment area (PA) for bacterial load.

### 3.3 Prevalence in worst-case sampling sites

Regarding active sampling methods and considering the wood exhibition area (WEA) and the payment area (PA) as the worst-case sampling sites within DIY stores, MAS-100 presented the highest fungal load (WEA-MEA: 250 CFU/m^3^|DG18: 385 CFU/m^3^; PA-MEA: 250 CFU/m^3^|DG18: 220 CFU/m^3^). The same scenario was seen in bacterial load (WEA-TSA: 240 CFU/m^3^; PA-TSA: 305 CFU/m^3^; Supplementary Figure S3).

Considering the load in Andersen six-stage cascade impactor, stage 5, which is equivalent to a viable airborne particle size of 1,1 μm, stood out from the other stages by obtaining a higher prevalence in 3 out of 4 sample media (WEA-TSA: 70.67 CFU/m^3^|DG18: 200.24 CFU/m^3^|DG18 37°C: 43.19 CFU/m^3^; Supplementary Figure S4).

Considering passive sampling methods, the one with the highest fungal contamination was surface swabs (WEA-MEA: 30000 CFU/m^2^|DG18: 40000 CFU/m^2^|DG18 37°C: 20000 CFU/m^2^; PA-MEA: 20000 CFU/m^2^|DG18: 10000 CFU/m^2^). Regarding bacterial contamination, surface swabs were also the one with the highest prevalence (WEA-TSA: 120000 CFU/m^2^; PA-TSA: 80000 CFU/m^2^; Supplementary Figure S5). In settled dust samples, DG18 presented the highest fungal counts (51 CFU/g), and TSA the highest bacterial counts (44 CFU/g; Supplementary Figure S6).

### 3.4 Fungal distribution by sampling method

**Figure 9:**
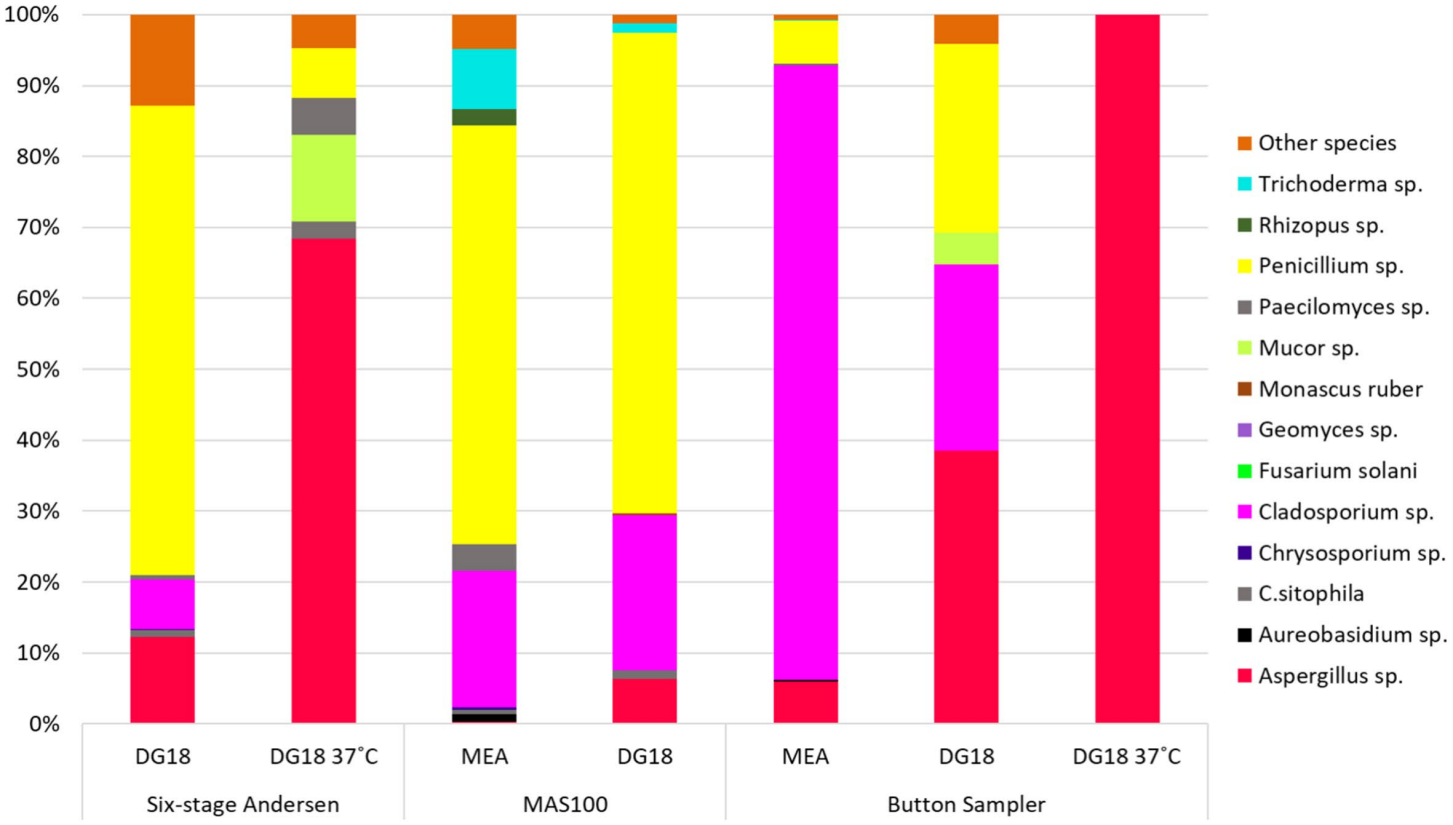
Species diversity on active sampling methods.

Taking into consideration the species found in active sampling methods *Aspergillu*s sp. presented the highest prevalence (DG18 37°C: Andersen six-stage cascade impactor (68.4%) and Button Sampler (100%) | DG18: Button Sampler (35.5%)), followed by P*enicillium* sp. and *Cladosporium* sp. (Figure 9).

**Figure 10:**
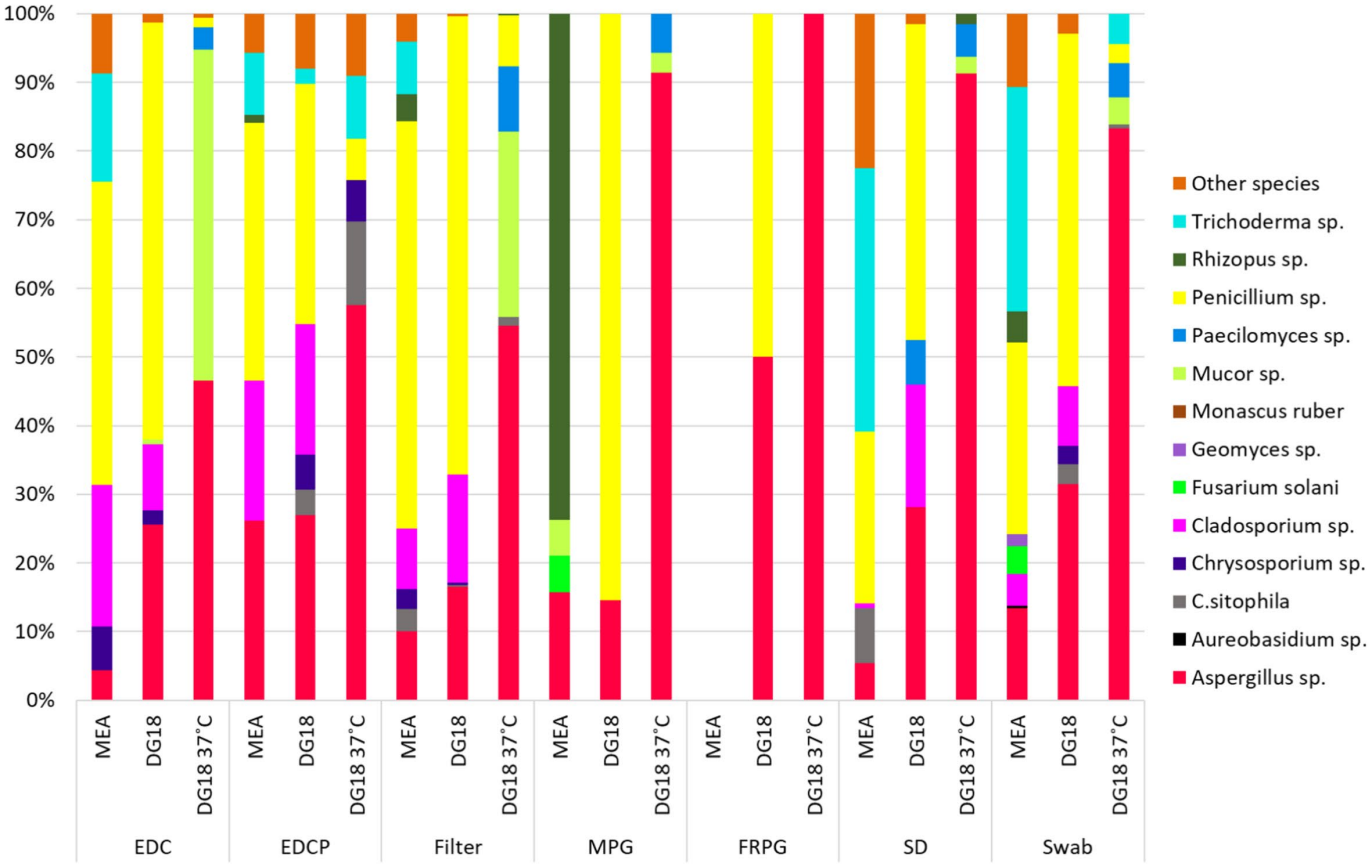
Species diversity on passive sampling methods.

Regarding passive sampling methods, *Penicillium* sp. presented the highest prevalence on DG18 (EDC: 60.78% | EDCP: 35.04% | Filters: 66.74% | MPG: 85.45% | FRPD: 50% | SD: 46.02% | Swabs: 51.30%) followed by *Aspergillus* sp. on DG18 incubated at 37°C (EDCP: 57.58% | Filters: 54.60% | MPG: 91.43% | FRPD: 100% | SD: 91.30% | Swabs: 83.33%). Two species from the order Mucorales also presented the highest prevalence in different passive sampling methods, such as *Mucor* sp. in EDC (DG18 37°C: 48.13%) and *Rhizopus* sp. in MPG (MEA: 73.68%; Figure 10).

### 3.5 Antifungal resistance profile

The screening of antifungal resistance in passive samples revealed that the most frequently observed fungal genera in SDA was *Penicillium* sp. (73.52%), followed by *Rhizopus* sp. (13.22%) and *C. sitophila* (12.16%).

On azole-supplemented media, only three fungal species grew, *C. sitophila* (ITZ: 98.06%; VCZ: 87.91%), *Mucor* sp. (ITZ: 1.75%; VCZ: 12.04%; PSZ: 99.23%), and *Rhizopus* sp. (ITZ: 0.20%; VCZ: 0.05%; PSZ: 0.77%; Supplementary Table S6). Only three sampling methods enabled fungal identification in azole-supplemented media, as follows: EDC [*Mucor* sp.: ITZ (9.83 CFU/m^2^/day); VCZ (29.57 CFU/m^2^/day)], SD [*Mucor* sp.: ITZ (8 CFU/g); VCZ (5 CFU/g), *Rhizopus* sp.: ITZ (2 CFU/g); VCZ (10 CFU/g)], and filters from the vacuum cleaner [*C.sitophila*: ITZ (1,000 CFU/m^2^); VCZ (18,500 CFU/m^2^), *Mucor* sp.: VCZ (2,500 CFU/m^2^)]. The *Aspergillus* genus was not found on azole-supplemented media. On SDA, two *Aspergillus* sections were found: *Fumigati* and *Circumdati*.

### 3.6 Mycotoxins contamination

Mycotoxins were detected in two sampling methods, in 11 settled dust samples (28.21%) and one filter from the vacuum cleaner (5.58%). Mycophenolic acid was the most detected mycotoxin in settled dust samples, found in 7 out of 39 samples (17.95%), with values ranging from <LOQ (11.7 ng/g) to 18.5 ng/g. This was followed by griseofulvin, detected in 5 out of 39 samples (12.82%) with values <LOQ (8.7 ng/g^1^), and aflatoxin G1, detected in 1 out of 39 samples (2.56%) with a value <LOQ (1.7 ng/g). It is important to highlight that in one of the settled dust samples, both aflatoxin G1 and griseofulvin were found with values <LOQ. On the filter from the vacuum cleaner, the only mycotoxin found was aflatoxin G1, with a value of 2.19 ng/g.

### 3.7 Cytotoxicity evaluation

Five serial 1:2 dilutions of samples’ extracts were tested against SK and A549 cells. Cellular viability was measured at 510 nm, and three cytotoxicity levels were defined based on sample dilution (Table 3). A higher number of samples induced more cytotoxicity in A549 cells (*N* = 97) than in SK cells (*N* = 44). Settled dust presented the highest cytotoxicity in both cell lines (SK: 75%; A549:50%), belonging these samples to the stores as a composite sample, followed by filters from the WEA (*n* = 1) and gloves from the WCA workers (*n* = 2) on SK cell line (12.5%). Followed by EDCP on the A549 cell line (25%) belonging these samples to the WCA (*n* = 1) and the PA (*n* = 2).

**Table 3:** Threshold toxicity level of filters, EDC, EDCP, masks, gloves, Coriolis, and settled dust in SK and A549 cells.

	SK				A549			
	High	Moderate	Low	(-)	High	Moderate	Low	(-)
Filters (*N* = 48)	1	0	3	44	0	8	34	6
EDC (*N* = 35)	0	1	5	29	1	6	10	18
EDCP (*N* = 38)	0	2	14	22	3	2	11	22
Masks (*N* = 6)	0	0	3	3	0	0	6	0
Gloves (*N* = 2)	1	1	0	0	2	0	0	0
Coriolis (*N* = 48)	0	0	0	48	0	0	1	47
Settled Dust (*N* = 13)	6	7	0	0	6	7	0	0
	8 (4.21%)	11 (5.79%)	25 (13.16%)	146 (76.84%)	12 (6.31%)	23 (12.11%)	62 (32.63%)	93 (48.95%)

A549, human alveolar epithelial cells; SK, human liver carcinoma cells; EDC, Electrostatic dust collector; High, IC50≥3rd dilution; Moderate, IC50 at 2nd dilution; Low, IC50 at 1st dilution; (-), no cytotoxicity.

### 3.8 Correlation analysis and biodiversity

For particulate matter, considering the strong correlations between the different particle sizes (Supplementary Figure S7), PCA was carried out, to reduce the information. From PCA, two components were obtained, C1 consisting of larger particles (PM_1.0_, PM_2.5_, PM_5.0_, and PM_10.0_), which retains 65% of the explained variance, and C2 consisting of particles of smaller size (PM_0.3_ and PM_0.5_), which retains 24.6% of the explained variance. In the following analyses, these two components were used.

Regarding active sampling methods, the following significant correlations were detected (Supplementary Table S7):

- MAS-100: (i) higher fungal contamination in MEA related with higher fungal contamination in DG18 (rS = 0.368, *p* = 0.004) and with higher bacterial contamination in TSA (rS = 0.417, *p* = 0.002); (ii) higher fungal contamination in DG18 related with higher bacterial contamination in TSA (rS = 0.377, *p* = 0.005), higher total particulate matter (TPM; rS = 0.364, p = 0.004) and higher larger particles concentration (rS = 0.330, *p* = 0.009); (iii) higher bacterial contamination in TSA with higher contamination in VRBA (rS = −0.574).

Regarding passive sampling methods, the following significant correlations were detected (Supplementary Table S7):

- Surface swabs: (i) greater fungal contamination in MEA with greater contamination in DG18 (rS = 0.591, *p* = 0.000) and DG18 37°C (rS = 0.438, *p* = 0.032), and with lower temperature (rS = −0.464, *p* = 0.003); (ii) higher fungal contamination in DG18 with higher contamination in DG18 37°C (rS = 0.537, *p* = 0.008); (iii) higher bacteria contamination in VRBA with higher relative humidity (rS = 0.894, *p* = 0.041).
- EDC: (i) higher fungal contamination in DG18 with higher values of azole resistance screening in SDA (rS = 0.458, *p* = 0.010).
- EDCP: (i) higher fungal contamination in MEA with lower bacteria contamination in TSA (rS = − 0.345, *p* = 0.040); (ii) higher fungal contamination in DG18 with higher contamination in DG18 37°C (rS = 0.501, *p* = 0.048).
- SD: (i) higher fungal contamination in MEA with higher contamination in DG18 (rS = 0.753, *p* = 0.003).
- Filters from vacuumed dust: (i) higher fungal contamination in MEA with higher contamination in DG18 (rS = 0.657, *p* = 0.000); (ii) DG18 37°C (rS = 0.598, *p* = 0.000) with higher values of azole resistance in SDA (rS = 0.531, *p* = 0.008) and in VCZ (rS = 0.777, *p* = 0.040); (iii) higher fungal contamination in DG18 with higher contamination in DG18 37°C (rS = 0.470, *p* = 0.004), higher bacteria contamination in TSA (rS = 0.363, *p* = 0.012) and higher values of azole resistance in SDA (rS = 0.722, *p* = 0.000); (iv) higher fungal contamination in DG18 37°C with higher values of azole resistance in SDA (rS = 0.704, *p* = 0.000) and lower concentration of smaller particles (rS = −0.378, *p* = 0.027); (v) higher bacteria contamination in TSA with higher values of azole resistance in SDA (rS = 0.413, *p* = 0.045), lower total particles concentration (rS = − 0.507, *p* = 0.000) and lower large particle concentration (rS = −0.442, *p* = 0.002).

Considering the correlation between particulate matter and the Andersen six-stage cascade impactor, particles of different dimensions were considered, as the intention was to explore the relationships with fungal contamination in DG18 with different pore sizes. The following correlations were detected (Supplementary Table S5): regarding fungal contamination in DG18, pore size 7 with particles PM_0.5_ (r_S_ = 0.397, *p* = 0.020) and PM_1.0_ (r_S_ = 0.411, *p* = 0.016); concerning fungal contamination in DG18 37°C: (i) pore size 1.1 and pore size 3.3 with temperature (r_S_ = 0.687, *p* = 0.001 and r_S_ = 0.420, *p* = 0.033, respectively); concerning bacterial contamination in TSA, pore size 0.65 with temperature (r_S_ = − 0.379, *p* = 0.036); and relating to bacterial contamination in VRBA: (i) pore size 2.1 with particles PM_10.0_ (r_S_ = 0.900, *p* = 0.037); (ii) pore size 3.3 and pore size 4.7 with TPM and with all types of particles except PM_0.3_ particles; (iii) pore size 7 with TPM and with all types of particles (Supplementary Table S8).

Regarding the biodiversity, the swabs presented, at the BWCA in MEA, the greatest biodiversity (Shannon Index (H) = 1.53, Simpson Index (D) = 4.28) followed by the filters, at the PA also in MEA (Shannon Index (H) = 1.35, Simpson Index (D) = 3.75; Supplementary Table S9).

## 4 Discussion

The sampling approach developed for studying microbial contamination in this occupational setting, relied on the complement of several active and passive sampling methods, aiming to overcome the constraints of all the methods and allow a proper characterization of the microbial risks to which these workers are exposed (23). It is well known that sampling protocols should include more than one type of sampling method. With stationary samplers, exposure to microorganisms is determined by averaging the airborne concentration over time at various sampling sites, while personal air sampling yields a more precise assessment of a worker’s exposure in the workplace (41, 42). Studies employing stationary sampling have not consistently demonstrated exposure-response associations as have data from personal sampling (41–43). As a result, data from stationary sampling must be interpreted by an experienced industrial hygienist using a more sophisticated exposure assessment protocol that may include various sampling strategies or analytical techniques (42).

Additionally, since there are several factors impacting microbial contamination indoors, passive sampling approaches allow a more complete assessment, since they can collect contamination over a longer period, thus covering all expected fluctuations (23, 44). Therefore, the combination of the two sampling methods offers a more precise approximation of the worker’s exposure in the workplace, justifying the sampling and analytical protocol applied in this study (41, 42).

Regarding the microbial load in each active sampling method, MAS-100 presented a higher fungal and bacterial load, which was expected when comparing the methods used, whereas, for passive sampling methods, surface swabs were the method with the highest values of total bacteria and fungi, followed by filters. *Aspergillus* sp., *Penicillium* sp., and *Cladosporium* sp. were the fungal species with higher prevalence among all sampling methods, which aligns with what was found in previous studies conducted in woodworking environments (10, 23, 45–47).

To allow prioritized interventions concerning the implementation of risk management measures, the worst-case exposure scenario regarding microbial load was identified. This was made based on the analysis of scientific and legal frames to pinpoint different sampling sites as the most critical workstations. In Portugal, as in most of the countries, there is only guidance concerning IAQ. While the wood exhibition area (WEA) was considered the worst sampling site for fungal load, the payment area (PA) was considered the worst for bacterial load. In addition, the (I/O) < 1 of the sampling sites supports the fact that the indoor bio-contaminants may have originated from the outdoor air (48, 49). These results may be justified due to the fact that although modern HVAC systems have features to improve indoor air quality, pollution in indoor spaces is caused by a variety of factors, such as indoor sources, pollutants entering from the outside through mechanical ventilation systems, and variations in microclimate (temperature, relative humidity). The concentration, size, and chemical makeup of the indoor particles as well as the penetration of outside particles are all influenced by these parameters (50–52). In all those stores, when applying these quantitative criteria (ratio I/O < 1) most of the stores were complying regarding the fungal load. As for the bacterial load, all stores were above the stipulated limit for the payment area (PA) suggesting that the bacterial load source was not outside but inside the stores.

Additionally, in this case, some species from the WHO priority list of fungal agents with pathogenic potential (53) were identified, namely *Aspergillus fumigatus* (which belongs to the critical group on the list) in all the stores, Mucorales (which belongs to the high-priority group on the list) in all the stores except for one, and also *Fusarium* sp. (which belongs to the high-priority group on the list) in two stores. It is important to note that there is no safe level of exposure to pathogenic microorganisms, so their presence should be null (54).

*Mucor* sp. and *Rhizopus* sp. (which belong to the order Mucorales) were identified in three azole-supplemented media during the screening of azole resistance. This is relevant because mucormycosis is an invasive fungal illness that can be destructive and fatal, and frequently linked to poor clinical results (55). Although Mucorales resistance to voriconazole and echinocandins was expected, as it is intrinsic to the species (55, 56), the identification of *Mucor* sp. and *Rhizopus* sp. in itraconazole and posaconazole raises concerns about cross-resistance and should be further investigated.

No *Aspergillus* section *Fumigati* isolates were recovered from azole-supplemented media, besides isolates recovered in MEA and DG18 incubated at both temperatures. This might be explained by nutrient competition with particular species that present high growth rates (e.g., Mucorales order), which may lead to the inhibition of fungal species with clinical relevance, such as *Aspergillus* section *Fumigati* (57).

Since fungi are not reliable indicators of the presence of mycotoxins, previous reports (44, 45) have already emphasized the significance of documenting mycotoxin presence in workplaces (45). This is mostly because not all fungi produce mycotoxins, which can remain in the environment long after fungi have been eradicated (46, 47). Furthermore, exposure to mycotoxins is often characterized by simultaneous exposure to multiple mycotoxins (44, 48), which represents an additional challenge for the risk assessment associated with this exposure because of the potential for mycotoxins to interact. This aspect results from several factors, including some fungi’s capacity to produce multiple mycotoxins (44, 45, 49). In DIY stores, three mycotoxins were found (mycophenolic acid, griseofulvin, and aflatoxin G1)., to the authors’ knowledge, This is the first report of mycotoxins in this occupational setting and the preventive steps that are taken to prevent fungal presence, are also useful to prevent mycotoxin contamination of the work setting, which includes cleaning surfaces frequently and properly (24). To prevent exposure to mycotoxins the same risk management measures to prevent exposure to PM should apply, since PM acts as a carrier for the workers’ respiratory system (58).

In woodworking environments, the wood dust that accumulates on surfaces is quite important since it can resuspend. Resuspension of dust is a common indoor source of airborne particulate matter inhalation exposure. It happens when previously deposited particles break free from surfaces like floors and re-enter interior air (59).

PM size affects deposition in the respiratory system and may cause several adverse health effects (60). Besides that, since airborne microorganisms tend to aggregate with particles of different sizes depending on the source, species, relative humidity, and mechanism of aerosolization (61–63), as previously mentioned, PM can act as a carrier of microorganisms and their metabolites for the respiratory system, facilitating exposure (8, 58). According to our data, PM contamination was higher in PM_10_, followed by PM_5.0_, indicating that wood dust can reach the gas exchange zone of the lung (PM_5.0_) and may cause illness by affecting the upper and bigger airways under the vocal cords (PM_10.0_) (8). PM sizes also influence the type of health effects that can be observed, namely that inflammatory responses are more strongly triggered by coarse particulate matter than by fine and ultrafine particulate matter (64, 65) which coincides with the correlations we obtained.

The correlation results showed some relevant information regarding the relation between fungal contamination in DG18 from MAS-100 and higher concentrations of PM_1.0_, PM_2.5_, PM_5.0_, and PM_10.0_, which implies that the microorganisms are being transported through airborne particles deep into the workers’ respiratory system affecting the gas exchange zone of the lungs. Additionally, considering that *Aspergillus* sp. had the highest prevalence in DG18 on both active and passive sampling methods. These results raise some concerns regarding workers’ exposure to potential pathogenic fungi. Regarding the cytotoxicity results, they showed that 50.05% of the samples had high to low cytotoxicity in A549 lung cells and 21.16% of the samples had high to low cytotoxicity in SK cells, suggesting that the lung cells were more sensitive to the contaminants in the samples. Exposing cells to the whole sample with mixed contaminants mimics the actual exposure scenario since lung cells are exposed directly to the pollutants (66). This pattern has been seen in other occupational settings, such as firefighters’ headquarters (24) and the waste industry (66). One important process that may be involved in cellular death is the multiple mycotoxin-induced toxicities (67). For instance, mycotoxins produced by *Fusarium* sp. (fumonisins) can induce cellular toxicity via mitochondrial stress and mitophagy (68). Other mycotoxins associated with *Aspergillus* section *Flavi* (aflatoxins) and *Aspergillus* section *Circumdati* (ochratoxin A) are also associated with cytotoxicity (69), thus highlighting the potential contribution of contaminants of biological origin to the observed cytotoxicity. Nevertheless, the cytotoxicity results from this study may be related to microbial contamination (bacteria, fungi, mycotoxins) and/or chemicals or particles (not assessed). Additionally, although the cytotoxicity assessment in environmental samples is crucial to identify and understand the correlation between all the environmental contaminants present in an occupational setting, since the mixture can have different toxicity from every contaminant assessed individually (70).

## 5 Conclusion

The multi-approach applied in this study, considering not only sampling methods but also the laboratory assays and different agents being measured (PM, microbial contamination, antifungal resistance, mycotoxins, and cytotoxicity), allowing a complete and robust analysis of this specific environment, thus enabling a more detailed risk assessment and prioritization of implementing risk management measures by Occupational Health Services. A limitation of this study is the lack of dust size identification in the personal air samples, as well as the fact that only 1 day was selected to apply most of the sampling methods, including PM monitoring which was only performed once in each sampling site for 5 min and this might be too short to capture the change in PM concentrations during the worst-case scenario.

Overall, some concerns were raised, such as (a) microbial contamination found did not comply with Portuguese IAQ legal requirements; (b) fungal exposure through inhalation underlines a possible risk factor for respiratory diseases; (c) the high prevalence of *Aspergillus* sp. incubated at 37°C which corroborates their pathogenic potential; (d) detection of mycotoxins presence for the first time in this setting; (e) high levels of cytotoxicity *in vitro,* particularly in lung epithelial cells; and (f) high contamination by PM acting as “transport vehicle” for microorganisms and mycotoxins into workers’ respiratory system.

Considering all the data available in this study, future research may be suggested: (a) conducting a more targeted evaluation based on size-specific particles since it can offer a more accurate understanding of the potential health effects associated with dust exposure; (b) an evaluation of the extent to which the *Aspergillus* section *Fumigati* isolates contribute to the total cytotoxicity and focus on the mutations found in this section isolate, specifically the ones that grew at 37°C; (c) evaluating the impact that the wood, once taken home by costumers, may act as a potential source of contamination of customers houses. This evaluation would enable a more comprehensive assessment of long-term exposure risks and associated health impacts resulting from the continued use of these materials in domestic environments; (d) developing a longitudinal study in this occupational setting to allow a longer sampling campaign that can overcome the limitations of cross-sectional studies, while offering valuable insights into exposure determinants and how these change over time.

## Data Availability

The original contributions presented in the study are included in the article/Supplementary material, further inquiries can be directed to the corresponding author.
