# Supplementary material for: Assessment of the microbial contamination in “Do It Yourself” (DIY) stores - a holistic approach to protect workers’ and consumers’ health
Source: Front Public Health. 2024 Oct 17;12:1483281. doi: 10.3389/fpubh.2024.1483281 (PMC11528695; doi:10.3389/fpubh.2024.1483281)
Supplement: Supplementary file 1 [file Table_1.docx]

**Supplementary Material**

**Table S1** - Walkthrough Survey applied in the DIY Stores

|  | **Yes** | **No** | **N/A** | **Answer/Comments** |
| --- | --- | --- | --- | --- |
| 1. **General Information** | | | | |
| - 1. Start of operations |  |  |  |  |
| - 1. Number of workers |  |  |  |  |
| - 1. Average number of customers per day |  |  |  |  |
| - 1. Busiest time |  |  |  |  |
| - 1. What machines are at the workstation (cutting area)? |  |  |  |  |
| - 1. Is there a storage area for the wood? |  |  |  |  |
| - 1. Does the store have natural ventilation? |  |  |  |  |
| - 1. Does the cutting area have natural ventilation? |  |  |  |  |
| - 1. Does the store have mechanical ventilation? If so, is it general or localised? |  |  |  |  |
| - 1. Does the cutting area have mechanical ventilation? If so, is it general or localised? |  |  |  |  |
| - 1. Is the cutting area isolated from the rest of the store? In what way? |  |  |  |  |
| 1. **Woods** (Derkyi, 2020; Kuznetsov & Kazansky, 2008; Sana et al., 2020; Straumfors et al., 2020)* | | | | |
| - 1. What types of wood are used? |  |  |  |  |
| - 1. Where does the wood come from? |  |  |  |  |
| - 1. Do you use solvents or varnishes? If so, which ones? |  |  |  |  |
| - 1. Do you use antifungal agents to treat the wood? If so, which ones? |  |  |  |  |
| 1. **Personal Protective Equipment** (Viegas, Dias, et al., 2020)* | | | | |
| - 1. What PPE workers use? |  |  |  |  |
| - 1. How often do they replace them? |  |  |  |  |
| - 1. Do they have a work uniform? |  |  |  |  |
| - 1. Do they have individualised lockers? |  |  |  |  |
| - 1. Do they have divided lockers (civilian clothes/work uniform)? |  |  |  |  |
| 1. **Cleaning and Disinfecting Procedures** (Dias et al., 2022; Viegas et al., 2021)* | | | | |
| - 1. Do you have procedures for cleaning and sanitizing work areas? If so, which ones? |  |  |  |  |
| - 1. How often do you clean and sanitise work areas? |  |  |  |  |
| - 1. What products are used? |  |  |  |  |
| 1. **Waste Management (Wood Shavings)** (Derkyi, 2020; Kuznetsov & Kazansky, 2008; Sana et al., 2020; Straumfors et al., 2020)* | | | | |
| - 1. What is the destination of the shavings? |  |  |  |  |
| - 1. How are the shavings packaged? |  |  |  |  |
| - 1. What is the process for extracting the shavings? |  |  |  |  |
| - 1. What is the process for chipping to its final destination? |  |  |  |  |

***References**

Derkyi, N. (2020). *Azoles for Renewable Energy Development and Wood Treatment*. https://doi.org/10.5772/intechopen.93472

Kuznetsov, Y. I., & Kazansky, L. P. (2008). Physicochemical aspects of metal protection by azoles as corrosion inhibitors. *Russian Chemical Reviews*, *77*(3), 219. https://doi.org/10.1070/RC2008v077n03ABEH003753

Sana, S., Reddy, V. G., Bhandari, S., Reddy, T. S., Tokala, R., Sakla, A. P., Bhargava, S. K., & Shankaraiah, N. (2020). Exploration of carbamide derived pyrimidine-thioindole conjugates as potential VEGFR-2 inhibitors with anti-angiogenesis effect. *European Journal of Medicinal Chemistry*, *200*, 112457. https://doi.org/10.1016/j.ejmech.2020.112457

Straumfors, A., Corbin, M., McLean, D., ‘t Mannetje, A., Olsen, R., Afanou, A., Daae, H.-L., Skare, Ø., Ulvestad, B., Laier Johnsen, H., Eduard, W., & Douwes, J. (2020). Exposure Determinants of Wood Dust, Microbial Components, Resin Acids and Terpenes in the Saw- and Planer Mill Industry. *Annals of Work Exposures and Health*, *64*(3), 282–296. https://doi.org/10.1093/annweh/wxz096

Viegas, C., Dias, M., Almeida, B., Aranha Caetano, L., Carolino, E., Quintal Gomes, A., Twarużek, M., Kosicki, R., Grajewski, J., Marchand, G., & Viegas, S. (2020). Are workers from waste sorting industry really protected by wearing Filtering Respiratory Protective Devices? The gap between the myth and reality. *Waste Management (New York, N.Y.)*, *102*, 856–867. https://doi.org/10.1016/j.wasman.2019.12.001

Viegas, C., Twarużek, M., Dias, M., Almeida, B., Carolino, E., Kosicki, R., Soszczyńska, E., Grajewski, J., Caetano, L. A., & Viegas, S. (2020). Assessment of the microbial contamination of mechanical protection gloves used on waste sorting industry: A contribution for the risk characterization. *Environmental Research*, *189*, 109881. https://doi.org/10.1016/j.envres.2020.109881

Table S2 – Sampling details

|  | **Sampling Method** | **Sampling Strategy Details** |
| --- | --- | --- |
| **Active Sampling Methods** | MAS-100 air tester | 200 L at a flow rate of 140 L/min. |
|  | Andersen six-stage cascade impactor | 28.3 L/min, for 9 min in each culture medium. |
|  | Coriolis μ air sampler | 300 L/min for 2 min with air samples being pumped into vials with 10 ml sterile phosphate-buffered saline (PBS - pH 7.4) with 0.05% Triton X100. |
|  | SKC Button Aerosol Sampler with a 0.8 µm 25 mm polycarbonate filter, connected to a SKC Universal air sampling pump | 4L/min for 2 hours. |
|  | Lighthouse Handheld Particle Counter | 2.83 L/min for 7 minutes (2 minutes to stabilize the equipment and 5 minutes for the sampling). |
| **Passive Sampling Methods** | Electrostatic dust collectors (EDC) | Placed in the sampling areas 1.5 m above the ground for 30 days. |
|  | Floor surface swabs | Swabbed using a 20 cm^2^ square stencil (disinfected between each sampling with a 70% alcohol solution). |
|  | E-cloths (EDCP) | Placed in three workers from different working areas with safety pins, during the entire work shift. |
|  | Settled dust and filter | Settled dust was vacuumed with a common vacuum cleaner (and respective filter). The dust was analysed as a composite sample of the whole store since the amount of dust was reduced due to the cleaning practices. |
|  | Filtering respiratory protection devices (FRPD) | FRPD (models N95, chemical mask with 6051 Dust Mask Filter and surgical masks were collected at the end of the work shift, but it is important to highlight that all of them had several days of use (1 to 4 days each). |
|  | Mechanical protection gloves (MPG) | MPG (model EN 388) was collected at the end of the work shift, but it is important to highlight that it had already 3 days of use. |

| **Sampling Method** | **Extraction for characterization of viable microbiota** | **Mycotoxins analysis*** |
| --- | --- | --- |
| Electrostatic dust collectors (EDC) | Washed with 10 mL of 0.1% Tween 80 saline (0.9% NaCl) solution (250 rpm, 30 min). | Raw extracts were diluted with an equal amount of water, mixed, centrifuged, and then injected into the LCMS/MS system for analysis. |
| Floor surface swabs | Washed with 1 mL of 0.1% Tween 80 saline (0.9% NaCl) solution (250 rpm, 30 min). |  |
| E-cloths (EDCP) | Washed with 10 mL of 0.1% Tween 80 saline (0.9% NaCl) solution (250 rpm, 30 min). |  |
| Settled dust | Washed with 9.1 mL/g of 0.1% Tween 80 saline (0.9% NaCl) solution (250 rpm, 30 min). |  |
| Filtering respiratory protection devices (FRPD) | Washed with 10 mL of 0.1% Tween 80 saline (0.9% NaCl) solution (250 rpm, 30 min). |  |
| Mechanical protection gloves (MPG) | Washed with 10 mL of 0.1% Tween 80 saline (0.9% NaCl) solution (250 rpm, 30 min). |  |
| Filter from vacuumed dust | Washed with 10 mL of 0.1% Tween 80 saline (0.9% NaCl) solution (250 rpm, 30 min). | Filter samples (0.2 g) were subjected to extraction with 2.0 ml of ACN: H2O: AcOH (79:20:1) for 60 minutes. |
| Filter from personal air sampler | Washed with 10 mL of 0.1% Tween 80 saline (0.9% NaCl) solution (250 rpm, 30 min). |  |
| Coriolis µ air sampler |  | For air samples (100 μl), a direct dilution of 1:7 (v/v) was conducted using a mixture of water and extraction solvent (acetonitrile (ACN): water (H2O): acetic acid (AcOH) (79:20:1)). |

Table S3 – Analysis details

***** Mycotoxins were separated on a chromatographic column Gemini NX-C18 (690 mm^2^, 3 μm) (Phenomenex, Torrance, CA, USA) with a mobile phase consisting of A: water + 5 mM ammonium acetate + 1% acetic acid, and B: methanol + 5 mM ammonium acetate + 1% acetic acid, at a flow rate of 0.75 ml/min. The injection volume was 7 μl. The concentration of mycotoxins was calculated using external calibration.

**Table S4** - Characterization of the 13 stores sampled and sampling methods employed

| **DIY stores** | **Building Age (years)** | **DIY area (m^2^)** | | **Type of ventilation** | | **Wood** | | **Average number of customers (per day)** | **Number of Workers** | **Cleaning frequency (per day)** | **Active sampling methods** | | | | | | **Passive sampling methods** | | | | | | | |
| --- | --- | --- | --- | --- | --- | --- | --- | --- | --- | --- | --- | --- | --- | --- | --- | --- | --- | --- | --- | --- | --- | --- | --- | --- |
|  |  | **Total** | **Cutting Zone** | **DIY store** | **Cutting Zone** | **Type** | **Origin** |  |  |  | **MAS-100** | **Andersen Six-stage** | **Button Sampler** | **Coriolis Air Sampler** | **Lighthouse** | **Surface swabs** | | **EDC** | | **EDCP** | **Settled dust** | **FRPD** | **MPG** |  |
| A | UNK | 8781 | 15 | Artificial | Artificial | Pine, Melamine, Plywood, OSB and MDF | Iberian Peninsula | 4000 | 300 | 1 | 21 | 24 | 2 | 4 | 5 | 4 | | 3 | | 3 | 4 | 1 | 1 |  |
| B | 18 | UNK | UNK | Artificial | Artificial | Pine, Chipboard, Plywood OSB, and MDF | Iberian Peninsula | UNK | 240 | 1 | 21 | 24 | 2 | 4 | 5 | 4 | | 3 | | 3 | 4 | 2 | 0 |  |
| C | UNK | UNK | UNK | Artificial | Artificial | Pine, Plywood, Particleboard, Oak and MDF | Iberian Peninsula | UNK | UNK | 1 | 21 | 24 | 2 | 4 | 5 | 4 | | 3 | | 3 | 4 | 1 | 0 |  |
| D | 2 | 2000 | 18 | Artificial | Artificial | Pine, Plywood, Particleboard, Oak and MDF | Iberian Peninsula | UNK | 32 | 1 | 21 | 24 | 2 | 4 | 5 | 4 | | 3 | | 3 | 4 | 0 | 0 |  |
| E | 26 | 3945 | 24 | Artificial | Artificial | Pine, Plywood, Particleboard, Oak and MDF | Iberian Peninsula | 1000 | 66 | 1 | 21 | 24 | 2 | 4 | 5 | 4 | | 3 | | 3 | 4 | 0 | 0 |  |
| F | 6 | UNK | UNK | Artificial | Artificial | Pine, Particleboard, Plywood and MDF | Iberian Peninsula | 700 | 37 | 1 | 21 | 24 | 2 | 4 | 5 | 4 | | 3 | | 3 | 4 | 0 | 0 |  |
| G | 1 | 4000 | 25 | Artificial | Artificial and natural | Pine, Chipboard, Melamine, Plywood, Natural OSB and MDF | International | 1000 | 82 | 3 | 21 | 24 | 2 | 4 | 5 | 4 | | 3 | | 3 | 4 | 0 | 0 |  |
| H | 13 | UNK | UNK | Artificial | Artificial | Pine, Plywood, Veneer, Bark, and MDF | Portugal and Northeastern countries | 1100 | 90 | 1 | 21 | 24 | 2 | 4 | 5 | 4 | | 3 | | 3 | 4 | 1 | 0 |  |
| I | UNK | UNK | 24 | Artificial | Artificial | Pine, Melamine, Plywood, Chipboard and MDF | Spain | UNK | UNK | 3 | 21 | 24 | 2 | 4 | 5 | 4 | | 3 | | 3 | 4 | 0 | 0 |  |
| J | 23 | 1932 | 12 | Artificial | Artificial | Pine and Chipboard | International | 800 | 30 | 1 | 21 | 24 | 2 | 4 | 5 | 4 | | 3 | | 3 | 4 | 0 | 0 |  |
| K | 5 | 2100 | 15 | Artificial | Artificial | Pine, Chipboard, Melamine and MDF | France and Poland | 900 | 25 | 1 | 21 | 24 | 2 | 4 | 5 | 4 | | 3 | | 3 | 4 | 0 | 0 |  |
| L | 27 | UNK | UNK | Artificial | Artificial | Pine, chipboard, Plywood, OSB and MDF | Portugal | 1500 | 114 | 1 | 21 | 24 | 2 | 4 | 5 | 4 | | 3 | | 3 | 4 | 0 | 0 |  |
| M | UNK | 14000 | 16 | Artificial and natural | Natural | Pine, Melamine, Chipboard, Plywood, OSB and MDF | UNK | UNK | UNK | 1 | 21 | 24 | 2 | 4 | 5 | 4 | | 3 | | 3 | 4 | 0 | 1 |  |
| **Total** | | | | | | | | | | | 273 | 312 | 26 | 52 | 65 | | 52 | | 39 | 39 | 52 | 5 | 2 | |

UNK – Unknown information

**Table S5** – The Limits of Detection (LOD) for each mycotoxin

| **Mycotoxin** | **Type of Sample** | |
| --- | --- | --- |
|  | **LOD for Coriolis** | **LOD for Filters, EDCP, EDC, Settled Dust, Gloves and Masks** |
| **15-Acetyldeoxynivalenol** | 7,8 | 8,1 |
| **3-Acetyldeoxynivalenol** | 5,4 | 5,7 |
| **Aflatoxin B1** | 0,6 | 0,5 |
| **Aflatoxin B2** | 0,5 | 0,5 |
| **Aflatoxin G1** | 0,5 | 0,5 |
| **Aflatoxin G2** | 0,9 | 0,7 |
| **Aflatoxin M1** | 0,9 | 0,8 |
| **aZearalanol** | 1,3 | 1,3 |
| **aZearalenol** | 0,7 | 1,0 |
| **bZearalanol** | 2,7 | 3,3 |
| **bZearalenol** | 1,8 | 1,9 |
| **Deepoxydeoxynivalenol** | 3,3 | 4,3 |
| **Deoxynivalenol** | 2,8 | 3,5 |
| **Deoxynivalenol-3-Glucoside** | 4,2 | 4,2 |
| **Diacetoxyscirpenol** | 1,9 | 2,3 |
| **Fumonisin B1** | 12,7 | 12,2 |
| **Fumonisin B2** | 7,2 | 7,5 |
| **Fumonisin B3** | 9,0 | 8,4 |
| **Fusarenon X** | 3,5 | 4,5 |
| **Gliotoxin** | 4,0 | 4,2 |
| **Griseofulvin** | 2,8 | 2,6 |
| **HT2** | 3,4 | 3,6 |
| **Mevinolin** | 2,4 | 2,2 |
| **Moniliformin** | 3,2 | 2,7 |
| **Monoacetoxyscirpenol** | 2,8 | 3,1 |
| **Mycophenolic acid** | 3,3 | 3,5 |
| **Neosolaniol** | 1,9 | 3,1 |
| **Nivalenol** | 4,3 | 4,3 |
| **Ochratoxin A** | 0,8 | 0,6 |
| **Ochratoxin B** | 1,3 | 1,5 |
| **Patulin** | 7,5 | 8,3 |
| **Roquefortine C** | 3,5 | 4,1 |
| **Sterigmatocystin** | 0,7 | 0,7 |
| **T2** | 1,0 | 0,9 |
| **T2 Tetraol** | 8,8 | 8,0 |
| **T2 Triol** | 6,0 | 5,5 |
| **Zearalanon** | 1,3 | 1,2 |
| **Zearalenon** | 0,5 | 0,8 |

**Methods Supplement S1** – Cytotoxicity Evaluation

Cells were maintained in Minimum Essential Medium Eagle (MEM) (Sigma-Aldrich, St. Louis, MI, USA) supplemented with an antibiotic solution (stock solution: 10,000 units of penicillin and 10 mg of streptomycin per mL in 0.9% NaCl (Sigma Aldrich)), and 5% fetal calf serum (Sigma-Aldrich) in a CO_2_-incubator (CB, BINDER GmbH, Tuttlingen, Germany) (5% CO_2_, 37˚C, 98% humidity). Cells were detached from the bottom of the culture vessel using 0.25% (w/v) Trypsin 0.53 mM EDTA and then suspended in the culture medium. Their number was determined using a cell counter (Scepter™ 2.0 Cell Counter, Merck Millipore, Burlington, MA, USA). Subsequently, 2.5 x 10^5^ cells were seeded per well of a 96-well microtiter plate. Cell suspensions (100 μl) were then incubated with the test samples in a 96-well plate for 48 h at 5% CO_2_, 37˚C, and a humid atmosphere. Then, MTT (3-(4,5-dimethylthiazol-2-yl)-2,5-diphenyltetrazolium bromide) solution (20 µL) was added, and plates were incubated for another 4 h. Subsequently, the supernatant was removed, and 100 µL dimethyl sulfoxide (DMSO) was added to each well. The cytotoxicity level was measured at 510 nm (ELISA LEDETECT 96, biomed Dr. Wieser GmbH; MikroWin 2013SC software). The lowest concentration, dropping absorption to <50% of cell metabolic activity (IC50), was considered the threshold toxicity level.


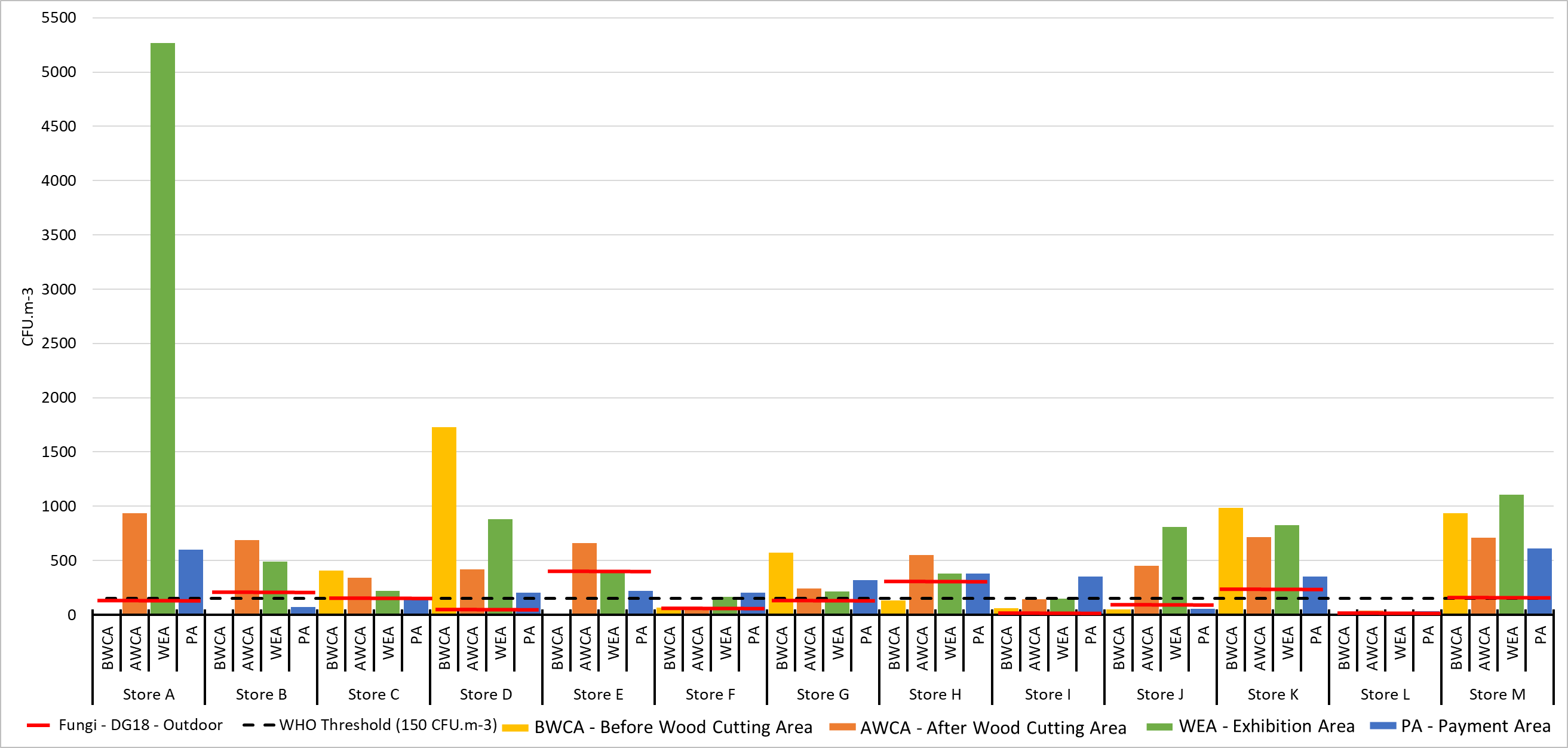

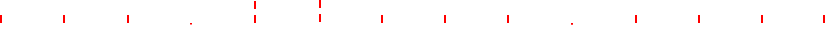


Figure S1 – Fungal contamination per sampling site in each store on DG18 (the red lines correspond to the concentration outdoor per store, the black non-continuous line corresponds to the WHO threshold)


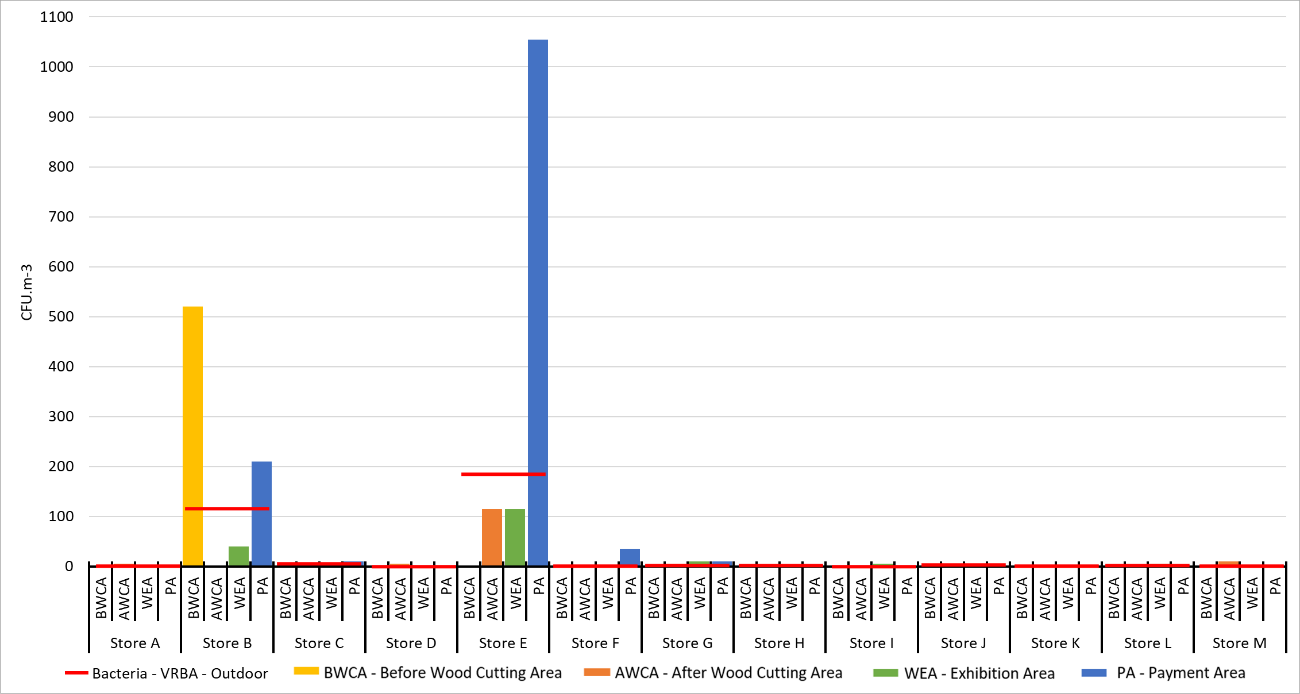

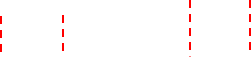


Figure S2 - Bacterial contamination per sampling site in each store on VRBA DG18 (the red lines correspond to the concentration outdoor per store)


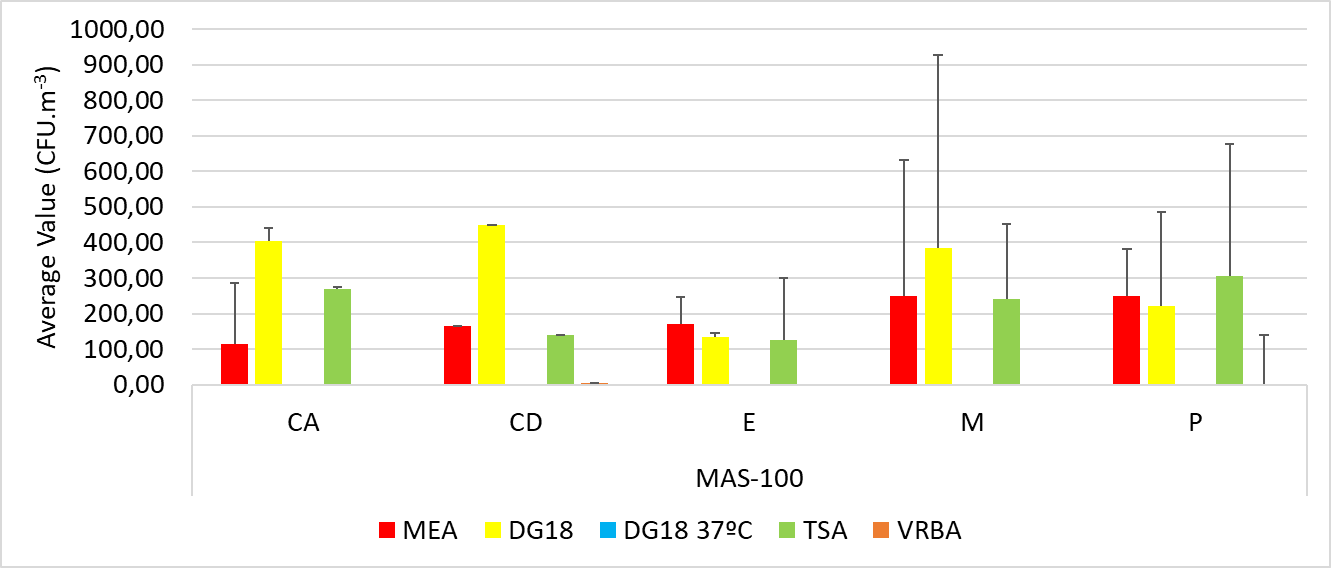


Figure S3 - Microbial contamination on MAS-100


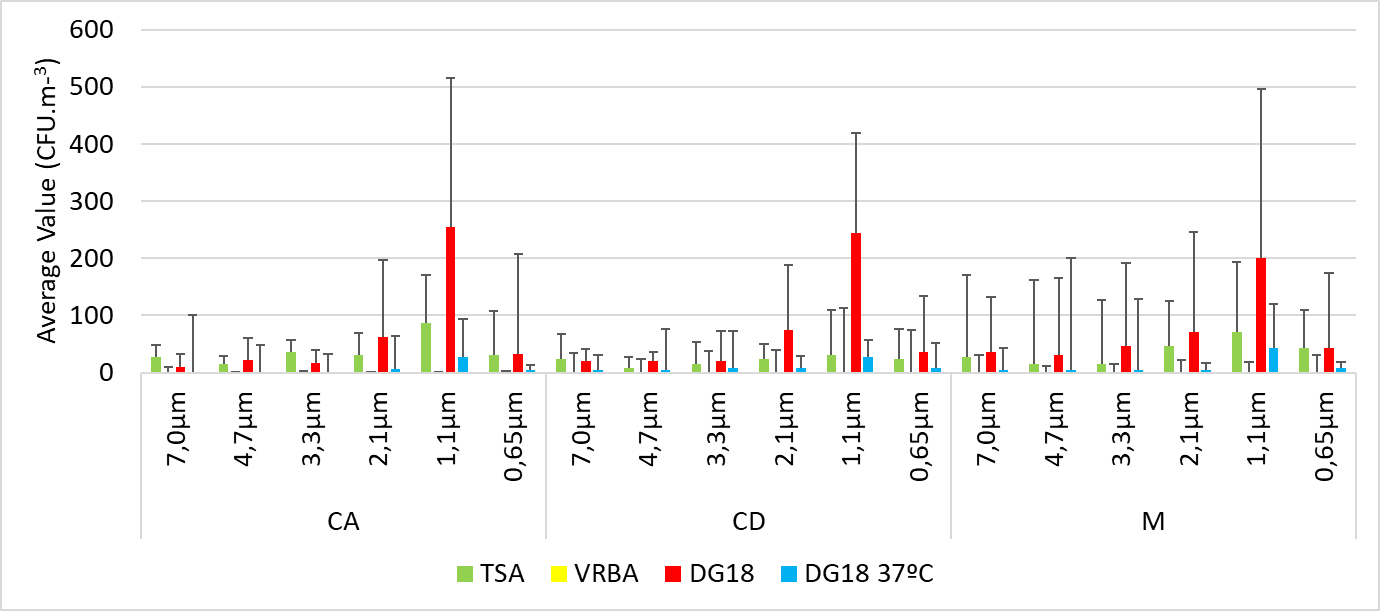


Figure S4 - Microbial contamination on Andersen six-stage


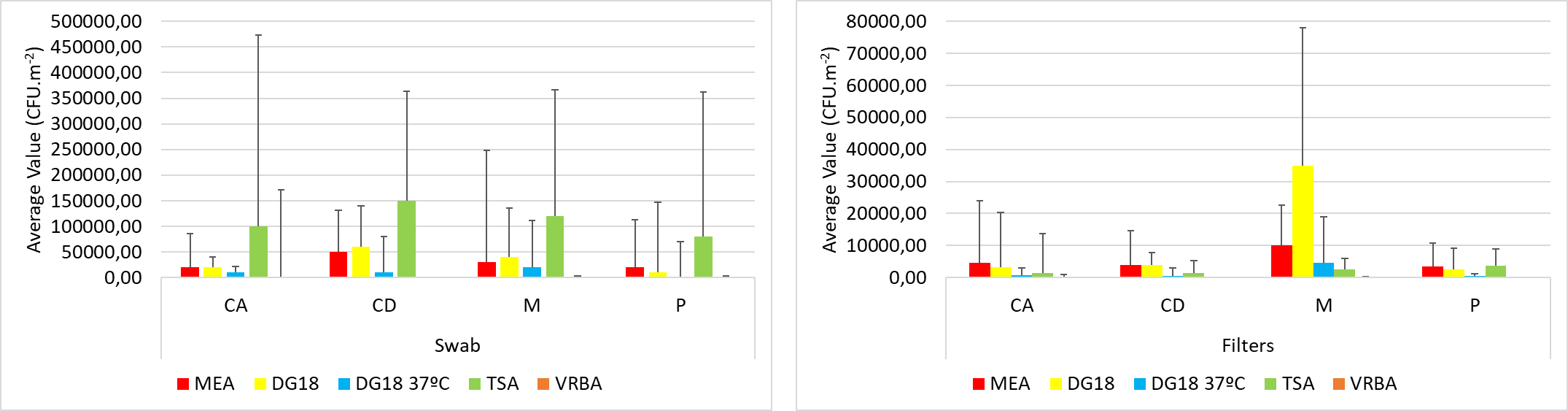


Figure S5 - Microbial contamination on surface swabs and filters from vacuum cleaner


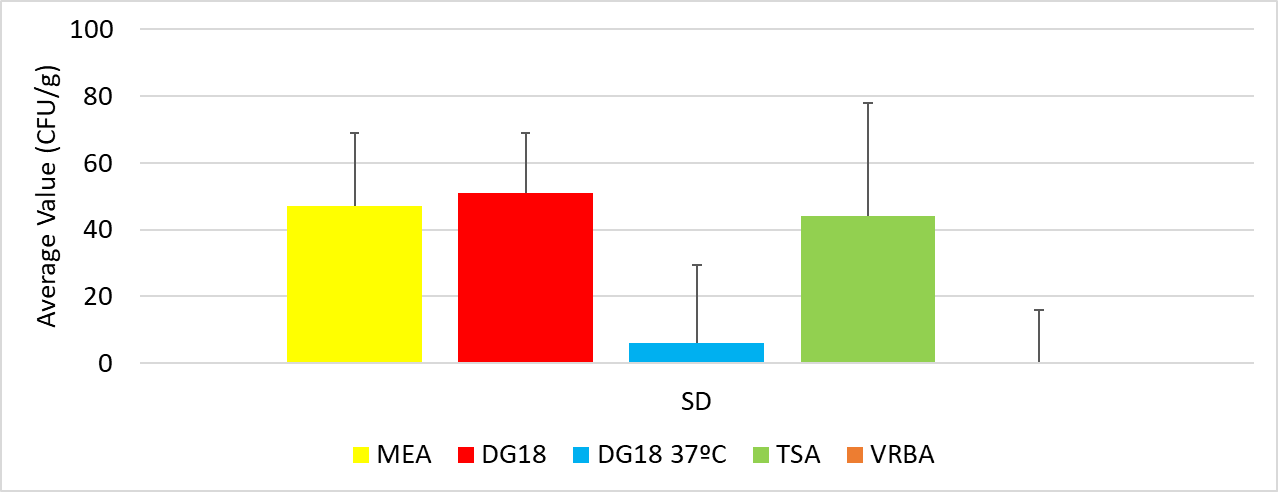


Figure S6 - Microbial contamination on settled dust

**Table S6** - Fungal distribution per azole-screening media

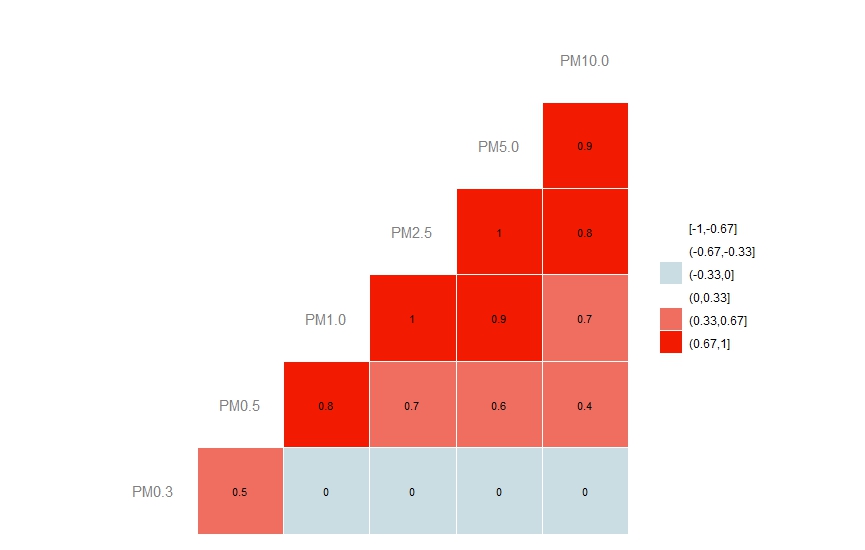


Figure S7 - Correlogram for particulate matter (rounded to 1 decimal place)

**Table S7 -** Study of the relationship between fungal contamination, bacterial contamination, azole resistance screening, particulate matter and environmental conditions in the sampling methods (MAS-100, button samplers, swab, EDC, EDCP, SD and filters) Spearman correlation coefficient results.

|  | | | | Fungi | | | | Bacteria | | | | Azole resistance screening | | | | | | | | Particulate Matter | | | | | | Environmental conditions | | | |
| --- | --- | --- | --- | --- | --- | --- | --- | --- | --- | --- | --- | --- | --- | --- | --- | --- | --- | --- | --- | --- | --- | --- | --- | --- | --- | --- | --- | --- | --- |
|  |  |  |  | DG18 | | DG18 37ºC | | TSA | | VRBA | | SDA | | ITZ | | VCZ | | PSZ | | TPM | | Smaller particles | | Large particles | | Relative Humidity (%) | | Temperature | |
| Sample method = MAS-100 | Fungi | MEA | 0.368^**^ | |  | | 0.417^**^ | | 0.085 | |  | |  | |  | |  | | -0.065 | | 0.146 | | -0.026 | | -0.057 | | -0.150 | |  |
|  |  | DG18 |  | |  | | 0.377^**^ | | 0.055 | |  | |  | |  | |  | | 0.364^**^ | | 0.082 | | 0.330^**^ | | -0.192 | | -0.057 | |  |
|  |  | DG18 37ºC |  | |  | |  | |  | |  | |  | |  | |  | |  | |  | |  | |  | |  | |  |
|  | Bacteria | TSA |  | |  | |  | | -0.574^*^ | |  | |  | |  | |  | | 0.056 | | -0.016 | | 0.010 | | 0.057 | | 0.096 | |  |
|  |  | VRBA |  | |  | |  | |  | |  | |  | |  | |  | | 0.087 | | -0.148 | | 0.053 | | -0.240 | | 0.430 | |  |
|  | Azole resistance screening | SDA |  | |  | |  | |  | |  | |  | |  | |  | |  | |  | |  | |  | |  | |  |
|  |  | ITZ |  | |  | |  | |  | |  | |  | |  | |  | |  | |  | |  | |  | |  | |  |
|  |  | VCZ |  | |  | |  | |  | |  | |  | |  | |  | |  | |  | |  | |  | |  | |  |
|  |  | PSZ |  | |  | |  | |  | |  | |  | |  | |  | |  | |  | |  | |  | |  | |  |
| Sample method = Button Samplers | Fungi | MEA | 0.171 | | 0.188 | | 0.064 | |  | | -0.632 | |  | |  | |  | |  | |  | |  | |  | |  | |  |
|  |  | DG18 |  | | 0.131 | | 0.055 | |  | | -0.632 | |  | |  | |  | |  | |  | |  | |  | |  | |  |
|  |  | DG18 37ºC |  | |  | | -0.078 | |  | |  | |  | |  | |  | |  | |  | |  | |  | |  | |  |
|  | Bacteria | TSA |  | |  | |  | |  | |  | |  | |  | |  | |  | |  | |  | |  | |  | |  |
|  |  | VRBA |  | |  | |  | |  | |  | |  | |  | |  | |  | |  | |  | |  | |  | |  |
|  | Azole resistance screening | SDA |  | |  | |  | |  | |  | |  | |  | |  | |  | |  | |  | |  | |  | |  |
|  |  | ITZ |  | |  | |  | |  | |  | |  | |  | |  | |  | |  | |  | |  | |  | |  |
|  |  | VCZ |  | |  | |  | |  | |  | |  | |  | |  | |  | |  | |  | |  | |  | |  |
|  |  | PSZ |  | |  | |  | |  | |  | |  | |  | |  | |  | |  | |  | |  | |  | |  |
| Sample method = Swab | Fungi | MEA | 0.591^**^ | | 0.438^*^ | | 0.318 | | -0.866 | |  | |  | |  | |  | | 0.222 | | 0.079 | | 0.144 | | -0.093 | | -0.464^**^ | |  |
|  |  | DG18 |  | | 0.537^**^ | | -0.042 | | -0.500 | |  | |  | |  | |  | | 0.204 | | -0.073 | | 0.225 | | 0.002 | | -0.253 | |  |
|  |  | DG18 37ºC |  | |  | | 0.205 | | -0.866 | |  | |  | |  | |  | | 0.209 | | -0.192 | | 0.249 | | 0.004 | | 0.160 | |  |
|  | Bacteria | TSA |  | |  | |  | | 0.775 | |  | |  | |  | |  | | -0.003 | | 0.177 | | -0.229 | | 0.180 | | -0.296 | |  |
|  |  | VRBA |  | |  | |  | |  | |  | |  | |  | |  | | -0.783 | | 0.224 | | -0.112 | | .894^*^ | | -0.112 | |  |
|  | Azole resistance screening | SDA |  | |  | |  | |  | |  | |  | |  | |  | |  | |  | |  | |  | |  | |  |
|  |  | ITZ |  | |  | |  | |  | |  | |  | |  | |  | |  | |  | |  | |  | |  | |  |
|  |  | VCZ |  | |  | |  | |  | |  | |  | |  | |  | |  | |  | |  | |  | |  | |  |
|  |  | PSZ |  | |  | |  | |  | |  | |  | |  | |  | |  | |  | |  | |  | |  | |  |
| Sample method = EDC | Fungi | MEA | 0.320 | | 0.278 | | 0.312 | |  | | 0.222 | |  | |  | |  | |  | |  | |  | |  | |  | |  |
|  |  | DG18 |  | | 0.019 | | 0.126 | |  | | 0.458^**^ | |  | |  | |  | |  | |  | |  | |  | |  | |  |
|  |  | DG18 37ºC |  | |  | | 0.171 | |  | | -0.178 | |  | |  | |  | |  | |  | |  | |  | |  | |  |
|  | Bacteria | TSA |  | |  | |  | |  | | 0.173 | |  | |  | |  | |  | |  | |  | |  | |  | |  |
|  |  | VRBA |  | |  | |  | |  | |  | |  | |  | |  | |  | |  | |  | |  | |  | |  |
|  | Azole resistance screening | SDA |  | |  | |  | |  | |  | |  | |  | |  | |  | |  | |  | |  | |  | |  |
|  |  | ITZ |  | |  | |  | |  | |  | |  | |  | |  | |  | |  | |  | |  | |  | |  |
|  |  | VCZ |  | |  | |  | |  | |  | |  | |  | |  | |  | |  | |  | |  | |  | |  |
|  |  | PSZ |  | |  | |  | |  | |  | |  | |  | |  | |  | |  | |  | |  | |  | |  |
| Sample method = EDCP | Fungi | MEA | 0.314 | | 0.162 | | -0.345^*^ | | -0.680 | | 0.300 | |  | |  | |  | |  | |  | |  | |  | |  | |  |
|  |  | DG18 |  | | 0.501^*^ | | 0.038 | | -0.616 | | 0.577 | |  | |  | |  | |  | |  | |  | |  | |  | |  |
|  |  | DG18 37ºC |  | |  | | 0.150 | |  | | -0.707 | |  | |  | |  | |  | |  | |  | |  | |  | |  |
|  | Bacteria | TSA |  | |  | |  | | -0.026 | | 0.617 | |  | |  | |  | |  | |  | |  | |  | |  | |  |
|  |  | VRBA |  | |  | |  | |  | |  | |  | |  | |  | |  | |  | |  | |  | |  | |  |
|  | Azole resistance screening | SDA |  | |  | |  | |  | |  | |  | |  | |  | |  | |  | |  | |  | |  | |  |
|  |  | ITZ |  | |  | |  | |  | |  | |  | |  | |  | |  | |  | |  | |  | |  | |  |
|  |  | VCZ |  | |  | |  | |  | |  | |  | |  | |  | |  | |  | |  | |  | |  | |  |
|  |  | PSZ |  | |  | |  | |  | |  | |  | |  | |  | |  | |  | |  | |  | |  | |  |
| Sample method = SD | Fungi | MEA | 0.753^**^ | | 0.507 | | 0.384 | | 0.500 | | 0.136 | |  | | -0.105 | | -0.200 | |  | |  | |  | |  | |  | |  |
|  |  | DG18 |  | | 0.405 | | 0.168 | | 0.500 | | 0.221 | | 0.500 | | 0.000 | | -0.103 | |  | |  | |  | |  | |  | |  |
|  |  | DG18 37ºC |  | |  | | 0.470 | | 0.866 | | -0.049 | |  | | 0.400 | | 0.700 | |  | |  | |  | |  | |  | |  |
|  | Bacteria | TSA |  | |  | |  | |  | | -0.423 | | -0.866 | |  | | -0.707 | |  | |  | |  | |  | |  | |  |
|  |  | VRBA |  | |  | |  | |  | | 0.500 | |  | |  | |  | |  | |  | |  | |  | |  | |  |
|  | Azole resistance screening | SDA |  | |  | |  | |  | |  | |  | | 0.200 | | 0.400 | |  | |  | |  | |  | |  | |  |
|  |  | ITZ |  | |  | |  | |  | |  | |  | |  | |  | |  | |  | |  | |  | |  | |  |
|  |  | VCZ |  | |  | |  | |  | |  | |  | |  | |  | |  | |  | |  | |  | |  | |  |
|  |  | PSZ |  | |  | |  | |  | |  | |  | |  | |  | |  | |  | |  | |  | |  | |  |
| Sample method = Filters | Fungi | MEA | ,657^**^ | | ,598^**^ | | 0,115 | | -0,866 | | ,531^**^ | |  | | ,777^*^ | |  | | -0,112 | | 0,007 | | -0,060 | | 0,268 | | 0,070 | |  |
|  |  | DG18 |  | | ,470^**^ | | ,363^*^ | | -0,866 | | ,722^**^ | |  | | 0,657 | |  | | -0,230 | | 0,205 | | -0,187 | | 0,215 | | -0,178 | |  |
|  |  | DG18 37ºC |  | |  | | -0,157 | | -0,866 | | ,704^**^ | |  | | 0,724 | |  | | 0,058 | | -,378^*^ | | -0,185 | | 0,198 | | 0,324 | |  |
|  | Bacteria | TSA |  | |  | |  | | 0,866 | | ,413^*^ | |  | | -0,289 | |  | | -,507^**^ | | 0,121 | | -0,442** | | -0,006 | | 0,052 | |  |
|  |  | VRBA |  | |  | |  | |  | |  | |  | |  | |  | | -0,866 | | 0,000 | | 0,000 | | 0,866 | | -0,866 | |  |
|  | Azole resistance screening | SDA |  | |  | |  | |  | |  | |  | | 0,775 | |  | | -0,277 | | -0,240 | | -0,113 | | -0,082 | | ,422^*^ | |  |
|  |  | ITZ |  | |  | |  | |  | |  | |  | |  | |  | |  | |  | |  | |  | |  | |  |
|  |  | VCZ |  | |  | |  | |  | |  | |  | |  | |  | | -0,031 | | -0,339 | | 0,139 | | 0,139 | | 0,563 | |  |
|  |  | PSZ |  | |  | |  | |  | |  | |  | |  | |  | |  | |  | |  | |  | |  | |  |
| *. Correlation is significant at the 0.05 level (2-tailed). **. Correlation is significant at the 0.01 level (2-tailed). Smaller particles - PM0.3 and PM0.5. Large particles - PM1.0 to PM10.0. | | | | | | | | | | | | | | | | | | | | | | | | | | | | | |

**Table S8** - Study of the relationship between fungal contamination and bacterial load obtained through the Andersen six-stages, with particulate matter and environmental conditions. Spearman correlation coefficient results

|  | | | Particulate matter | | | | | | | Environmental conditions | |  |
| --- | --- | --- | --- | --- | --- | --- | --- | --- | --- | --- | --- | --- |
|  |  |  |  |  |  |  |  |  |  |  |  |  |
|  | Culture Medium | Pore size | TPM | PM00.3 | PM0.5 | PM1.0 | PM2.5 | PM5.0 | PM10.0 | Relative Humidity (%) | Temperature |  |
| Fungi | DG18 | 0.65 | 0.294 | 0.170 | 0.296 | 0.310 | 0.188 | 0.182 | 0.205 | -0.291 | -0.170 |  |
|  |  | 1.1 | -0.050 | 0.000 | 0.108 | -0.005 | -0.124 | -0.123 | -0.057 | -0.310 | -0.132 |  |
|  |  | 2.1 | 0.262 | -0.073 | 0.272 | 0.235 | 0.166 | 0.185 | 0.196 | -0.240 | -0.056 |  |
|  |  | 3.3 | 0.131 | -0.124 | 0.159 | 0.072 | 0.035 | 0.060 | 0.159 | -0.149 | -0.013 |  |
|  |  | 4.7 | 0.165 | 0.185 | 0.304 | 0.122 | 0.093 | 0.071 | 0.122 | -0.047 | -0.142 |  |
|  |  | 7 | 0.103 | 0.118 | 0.397^*^ | 0.411^*^ | 0.256 | 0.132 | 0.028 | -0.041 | 0.024 |  |
|  | DG18 37ºC | 0.65 | 0.055 | -0.135 | -0.253 | 0.007 | 0.140 | 0.114 | 0.140 | 0.140 | 0.377 |  |
|  |  | 1.1 | -0.307 | -0.263 | -0.299 | -0.229 | -0.194 | -0.287 | -0.291 | -0.048 | 0.687^**^ |  |
|  |  | 2.1 | -0.166 | -0.152 | -0.190 | -0.138 | -0.079 | -0.138 | -0.177 | -0.134 | 0.410 |  |
|  |  | 3.3 | -0.022 | -0.202 | -0.097 | -0.037 | 0.035 | 0.041 | -0.002 | -0.041 | 0.420^*^ |  |
|  |  | 4.7 | -0.013 | -0.177 | -0.255 | -0.075 | 0.026 | 0.025 | -0.006 | 0.115 | 0.170 |  |
|  |  | 7 | 0.021 | -0.080 | -0.164 | 0.073 | 0.030 | -0.014 | 0.044 | -0.053 | 0.208 |  |
| Bacteria | TSA | 0.65 | 0.109 | 0.003 | -0.210 | -0.036 | 0.078 | 0.094 | 0.105 | 0.118 | -0.379^*^ |  |
|  |  | 1.1 | 0.196 | -0.061 | -0.146 | 0.088 | 0.199 | 0.127 | 0.123 | 0.124 | 0.022 |  |
|  |  | 2.1 | 0.044 | 0.192 | -0.038 | 0.020 | 0.064 | 0.045 | 0.071 | 0.126 | -0.063 |  |
|  |  | 3.3 | -0.095 | 0.037 | -0.050 | 0.045 | -0.031 | -0.101 | -0.139 | 0.037 | 0.136 |  |
|  |  | 4.7 | -0.244 | 0.002 | -0.028 | -0.069 | -0.179 | -0.232 | -0.310 | 0.082 | -0.043 |  |
|  |  | 7 | -0.153 | -0.145 | -0.126 | 0.033 | -0.052 | -0.139 | -0.238 | 0.194 | 0.352 |  |
|  | VRBA | 0.65 | 0.200 | -0.400 | 0.200 | 0.200 | 0.200 | 0.200 | 0.200 | 0.800 | -0.872 |  |
|  |  | 1.1 |  | 0.600 | 0.800 |  |  |  | 0.800 | 0.200 | -0.211 |  |
|  |  | 2.1 | 0.800 | -0.300 | 0.600 | 0.600 | 0.800 | 0.800 | 0.900^*^ | 0.100 | 0.667 |  |
|  |  | 3.3 | 0.806^**^ | -0.068 | 0.923^**^ | 0.948^**^ | 0.849^**^ | 0.849^**^ | 0.769^**^ | -0.166 | 0.422 |  |
|  |  | 4.7 | 0.986^**^ | 0.319 | 0.899^*^ | 0.986^**^ | 0.986^**^ | 0.986^**^ | 0.754 | -0.319 | -0.030 |  |
|  |  | 7 | 0.886^*^ | 0.829^*^ | 0.943^**^ | 0.943^**^ | 0.943^**^ | 0.886^*^ | 0.886^*^ | -0.429 | -0.441 |  |
| *. Correlation is significant at the 0.05 level (2-tailed). **. Correlation is significant at the 0.01 level (2-tailed). | | | | | | | | | | | |  |

**Table S9** – Biodiversity: Shannon and Simpson indices

| Matrice | Sampling Place | Culture media | Genera/Species | Culture Media (CFU/m-2/-3) | Shannon Index (H) | Simpson Index (D) |  | Matrice | Sampling Place | | Culture media | Genera/Species | Culture Media (CFU/m-2/-3) | Shannon Index (H) | Simpson Index (D) |
| --- | --- | --- | --- | --- | --- | --- | --- | --- | --- | --- | --- | --- | --- | --- | --- |
|  |  |  |  |  |  |  |  |  |  |  |  |  |  |  |  |
| BS | C | MEA | *Aureobasidium* sp. | 4,17 | 0,38 | 1,21 |  | EDCP | C | | MEA | *Cladosporium* sp. | 1,19 | 1,31 | 3,46 |
|  | C | MEA | *Cladosporium* sp. | 1000,00 |  |  |  |  | C | | MEA | *Penicillium* sp. | 1,58 |  |  |
|  | C | MEA | *Penicillium* sp. | 22,92 |  |  |  |  | C | | MEA | *Trichoderma* sp. | 0,79 |  |  |
|  | C | MEA | *Aspergillus* sp. | 77,08 |  |  |  |  | C | | MEA | *Aspergillus* sp. | 2,37 |  |  |
|  | Total | | | 1104,17 |  |  |  |  | Total | | | | 5,93 |  |  |
|  | CL | MEA | *Penicillium* sp. | 2,08 | 0,69 | 2,00 |  |  | CL | | MEA | *Rhizopus* sp. | 0,40 | 0,00 | 1,00 |
|  | CL | MEA | *Trichoderma* sp. | 2,08 |  |  |  |  | Total | | | | 0,40 |  |  |
|  | Total | | | 4,17 |  |  |  |  | M | | MEA | *Cladosporium* sp. | 3,95 | 1,22 | 3,18 |
|  | P | MEA | *Cladosporium* sp. | 475,00 | 0,61 | 1,46 |  |  | M | | MEA | *Penicillium* sp. | 6,32 |  |  |
|  | P | MEA | *Paecilomyces* sp. | 4,17 |  |  |  |  | M | | MEA | *Trichoderma* sp. | 0,79 |  |  |
|  | P | MEA | *Penicillium* sp. | 79,17 |  |  |  |  | M | | MEA | *Aspergillus* sp. | 5,14 |  |  |
|  | P | MEA | *Aspergillus* sp. | 25,00 |  |  |  |  | Total | | | | 16,21 |  |  |
|  | Total | | | 583,33 |  |  |  |  | P | | MEA | *Cladosporium* sp. | 1,98 | 1,24 | 2,99 |
|  | C | DG18 | *Cladosporium* sp. | 85,42 | 1,11 | 2,64 |  |  | P | | MEA | *Penicillium* sp. | 5,14 |  |  |
|  | C | DG18 | *Mucor* sp. | 12,50 |  |  |  |  | P | | MEA | *Trichoderma* sp. | 1,58 |  |  |
|  | C | DG18 | *Penicillium* sp. | 68,75 |  |  |  |  | P | | MEA | *Aspergillus* sp. | 1,58 |  |  |
|  | C | DG18 | *Aspergillus* sp. | 189,58 |  |  |  |  | Total | | | | 10,28 |  |  |
|  | Total | | | 356,25 |  |  |  |  | C | | DG18 | *Cladosporium* sp. | 4,74 | 1,19 | 2,88 |
|  | P | DG18 | *Cladosporium* sp. | 62,50 | 1,19 | 2,95 |  |  | C | | DG18 | *Penicillium* sp. | 8,30 |  |  |
|  | P | DG18 | *Mucor* sp. | 12,50 |  |  |  |  | C | | DG18 | *Trichoderma* sp. | 1,19 |  |  |
|  | P | DG18 | *Penicillium* sp. | 81,25 |  |  |  |  | C | | DG18 | *Aspergillus* sp. | 2,77 |  |  |
|  | P | DG18 | *Aspergillus* sp. | 27,08 |  |  |  |  | Total | | | | 17,00 |  |  |
|  | Total | | | 183,33 |  |  |  |  | CL | | DG18 | *Aspergillus* sp. | 0,40 | 0,00 | 1,00 |
|  | C | DG18 37ºC | *Aspergillus* sp. | 33,33 | 0,00 | 1,00 |  |  | Total | | | | 0,40 |  |  |
|  | Total | | | 33,33 |  | 1,00 |  |  | M | | DG18 | *Chrysosporium* sp. | 2,77 | 1,32 | 3,52 |
|  | CL | DG18 37ºC | *Aspergillus* sp. | 2,08 | 0,00 | 1,00 |  |  | M | | DG18 | *Cladosporium* sp. | 4,35 |  |  |
|  | Total | | | 2,08 |  | 1,00 |  |  | M | | DG18 | *Penicillium* sp. | 6,72 |  |  |
|  | P | DG18 37ºC | *Aspergillus* sp. | 37,50 | 0,00 | 1,00 |  |  | M | | DG18 | *Aspergillus* sp. | 7,91 |  |  |
|  | Total | | | 37,50 |  |  |  |  | Total | | | | 21,74 |  |  |
|  | C | SAB | *Cladosporium* sp. | 6,25 | 0,56 | 1,60 |  |  | P | | DG18 | *C.sitophila* | 1,98 | 1,29 | 3,39 |
|  | C | SAB | *Penicillium* sp. | 18,75 |  |  |  |  | P | | DG18 | *Cladosporium* sp. | 1,19 |  |  |
|  | Total | | | 25,00 |  |  |  |  | P | | DG18 | *Penicillium* sp. | 3,95 |  |  |
|  | P | SAB | *Cladosporium* sp. | 35,42 | 0,21 | 1,12 |  |  | P | | DG18 | *Aspergillus* sp. | 3,56 |  |  |
|  | P | SAB | *Mucor* sp. | 2,08 |  |  |  |  | Total | | | | 10,67 |  |  |
|  | Total | | | 37,50 |  |  |  |  | C | | DG18 37ºC | *C.sitophila* | 0,79 | 1,15 | 2,58 |
| EDC | C | MEA | *Chrysosporium* sp. | 198,06 | 1,21 | 2,93 |  |  | C | | DG18 37ºC | *Chrysosporium* sp. | 0,79 |  |  |
|  | C | MEA | *Cladosporium* sp. | 542,64 |  |  |  |  | C | | DG18 37ºC | *Trichoderma* sp. | 0,79 |  |  |
|  | C | MEA | *Penicillium* sp. | 864,90 |  |  |  |  | C | | DG18 37ºC | *Aspergillus* sp. | 3,16 |  |  |
|  | C | MEA | *Trichoderma* sp. | 211,97 |  |  |  |  | Total | | | | 5,53 |  |  |
|  | Total | | | 1817,57 |  |  |  |  | CL | | DG18 37ºC | *Aspergillus* sp. | 0,79 | 0,00 | 1,00 |
|  | M | MEA | *Chrysosporium* sp. | 53,23 | 1,22 | 3,08 |  |  | Total | | | | 0,79 |  |  |
|  | M | MEA | *Cladosporium* sp. | 112,71 |  |  |  |  | M | | DG18 37ºC | *C.sitophila* | 0,40 | 0,68 | 1,59 |
|  | M | MEA | *Penicillium* sp. | 283,05 |  |  |  |  | M | | DG18 37ºC | *Penicillium* sp. | 0,40 |  |  |
|  | M | MEA | *Trichoderma* sp. | 258,03 |  |  |  |  | M | | DG18 37ºC | *Aspergillus* sp. | 2,77 |  |  |
|  | Total | | | 707,02 |  |  |  |  | Total | | | | 3,56 |  |  |
|  | P | MEA | *Cladosporium* sp. | 157,30 | 1,18 | 2,68 |  |  | P | | DG18 37ºC | *C.sitophila* | 0,40 | 1,33 | 3,57 |
|  | P | MEA | *Penicillium* sp. | 593,29 |  |  |  |  | P | | DG18 37ºC | *Penicillium* sp. | 0,40 |  |  |
|  | P | MEA | *Trichoderma* sp. | 147,96 |  |  |  |  | P | | DG18 37ºC | *Trichoderma* sp. | 0,40 |  |  |
|  | P | MEA | *Aspergillus* sp. | 173,03 |  |  |  |  | P | | DG18 37ºC | *Aspergillus* sp. | 0,79 |  |  |
|  | Total | | | 1071,57 |  |  |  |  | Total | | | | 1,98 |  |  |
|  | C | DG18 | *Chrysosporium* sp. | 32,70 | 0,95 | 2,33 |  |  | C | | SDA | *Penicillium* sp. | 0,79 | 0,00 | 1,00 |
|  | C | DG18 | *Cladosporium* sp. | 219,29 |  |  |  |  | Total | | | | 0,79 |  |  |
|  | C | DG18 | *Penicillium* sp. | 1497,94 |  |  |  |  | CL | | SDA | Aspergillus sp. | 0,40 | 0,00 | 1,00 |
|  | C | DG18 | *Aspergillus* sp. | 1221,22 |  |  |  |  | Total | | | | 0,40 |  |  |
|  | Total | | | 2971,15 |  |  |  |  | M | | SDA | *Aspergillus* sp. | 0,79 | 0,00 | 1,00 |
|  | M | DG18 | *Chrysosporium* sp. | 127,84 | 0,73 | 1,55 |  |  | Total | | | | 0,79 |  |  |
|  | M | DG18 | *Cladosporium* sp. | 214,32 |  |  |  |  | P | | SDA | *Penicillium* sp. | 3,95 | 0,00 | 1,00 |
|  | M | DG18 | *Penicillium* sp. | 2169,10 |  |  |  |  | Total | | | | 3,95 |  |  |
|  | M | DG18 | *Aspergillus* sp. | 218,94 |  |  |  | Filter | CA | | MEA | *Cladosporium* sp. | 15000,00 | 0,80 | 1,69 |
|  | Total | | | 2730,19 |  |  |  |  | CA | | MEA | *Penicillium* sp. | 82000,00 |  |  |
|  | P | DG18 | *Cladosporium* sp. | 310,32 | 1,08 | 2,56 |  |  | CA | | MEA | *Rhizopus* sp. | 5000,00 |  |  |
|  | P | DG18 | *Mucor* sp. | 48,57 |  |  |  |  | CA | | MEA | *Aspergillus* sp. | 7000,00 |  |  |
|  | P | DG18 | *Penicillium* sp. | 1039,24 |  |  |  |  | Total | | | | 109000,00 |  |  |
|  | P | DG18 | *Aspergillus* sp. | 545,04 |  |  |  |  | CD | | MEA | *Chysosporium* sp. | 13000,00 | 1,02 | 2,18 |
|  | Total | | | 1943,16 |  |  |  |  | CD | | MEA | *Cladosporium* sp. | 6000,00 |  |  |
|  | C | DG18 37ºC | *Mucor* sp. | 560,43 | 0,82 | 2,07 |  |  | CD | | MEA | *Penicillium* sp. | 68000,00 |  |  |
|  | C | DG18 37ºC | *Paecilomyces* sp. | 37,98 |  |  |  |  | CD | | MEA | *Aspergillus* sp. | 19500,00 |  |  |
|  | C | DG18 37ºC | *Penicillium* sp. | 8,28 |  |  |  |  | Total | | | | 106500,00 |  |  |
|  | C | DG18 37ºC | *Aspergillus* sp. | 795,24 |  |  |  |  | M | | MEA | *C.sitophila* | 15000,00 | 0,98 | 2,14 |
|  | Total |  |  | 1401,93 |  |  |  |  | M | | MEA | *Cladosporium* sp. | 5500,00 |  |  |
|  | M | DG18 37ºC | *Mucor* sp. | 405,89 | 0,83 | 2,12 |  |  | M | | MEA | *Penicillium* sp. | 95500,00 |  |  |
|  | M | DG18 37ºC | *Paecilomyces* sp. | 14,85 |  |  |  |  | M | | MEA | *Trichoderma* sp. | 34500,00 |  |  |
|  | M | DG18 37ºC | *Penicillium* sp. | 10,48 |  |  |  |  | Total | | | | 150500,00 |  |  |
|  | M | DG18 37ºC | *Aspergillus* sp. | 335,38 |  |  |  |  | P | | MEA | *Cladosporium* sp. | 13000,00 | 1,35 | 3,75 |
|  | Total | | | 766,61 |  |  |  |  | P | | MEA | *Penicillium* sp. | 23500,00 |  |  |
|  | P | DG18 37ºC | *Mucor* sp. | 410,49 | 0,94 | 2,16 |  |  | P | | MEA | *Rhizopus* sp. | 13000,00 |  |  |
|  | P | DG18 37ºC | *Paecilomyces* sp. | 39,32 |  |  |  |  | P | | MEA | *Aspergillus* sp. | 19000,00 |  |  |
|  | P | DG18 37ºC | *Penicillium* sp. | 23,13 |  |  |  |  | Total | | | | 68500,00 |  |  |
|  | P | DG18 37ºC | *Aspergillu*s sp. | 202,44 |  |  |  |  | CA | | DG18 | *Chrysosporium* sp. | 2000,00 | 0,90 | 2,16 |
|  | Total | | | 675,37 |  |  |  |  | CA | | DG18 | *Cladosporium* sp. | 35500,00 |  |  |
|  | C | SAB | *C.sitophila* sp. | 50,68 | 0,37 | 1,19 |  |  | CA | | DG18 | *Penicillium* sp. | 55000,00 |  |  |
|  | C | SAB | *Cladosporium* sp. | 107,61 |  |  |  |  | CA | | DG18 | *Aspergillus* sp. | 4000,00 |  |  |
|  | C | SAB | *Penicillium* sp. | 1929,45 |  |  |  |  | Total | | | | 96500,00 |  |  |
|  | C | SAB | *Rhizopus* sp. | 22,47 |  |  |  |  | CD | | DG18 | Cladosporium sp. | 5000,00 | 1,02 | 2,40 |
|  | Total | | | 2110,20 |  |  |  |  |  |  |  |  |  |  |  |
|  | M | SAB | C.sitophila | 161,68 | 0,79 | 1,76 |  |  | CD | | DG18 | *Penicillium* sp. | 33000,00 |  |  |
|  | M | SAB | *Mucor* sp. | 33,72 |  |  |  |  | CD | | DG18 | *Aspergillus* sp. | 24500,00 |  |  |
|  | M | SAB | *Penicillium* sp. | 546,76 |  |  |  |  | Total | | | | 62500,00 |  |  |
|  | M | SAB | *Rhizopus* sp. | 16,55 |  |  |  |  | M | | DG18 | C.sitophila | 1500,00 | 0,78 | 1,75 |
|  | Total | | | 758,72 |  |  |  |  | M | | DG18 | *Cladosporium* sp. | 62000,00 |  |  |
|  | P | SAB | *C.sitophila* | 101,85 | 0,82 | 1,90 |  |  | M | | DG18 | *Penicillium* sp. | 340500,00 |  |  |
|  | P | SAB | *Mucor* sp. | 182,90 |  |  |  |  | M | | DG18 | *Aspergillus* sp. | 60500,00 |  |  |
|  | P | SAB | *Penicillium* sp. | 632,46 |  |  |  |  | Total | | | | 464500,00 |  |  |
|  | Total | | | 917,21 |  |  |  |  | P | | DG18 | *Cladosporium* sp. | 6500,00 | 0,95 | 2,40 |
|  | M | ITZ | *Mucor* sp. | 9,83 | 0,00 | 1,00 |  |  | P | | DG18 | *Penicillium* sp. | 31000,00 |  |  |
|  | Total | | | 9,83 |  |  |  |  | P | | DG18 | *Aspergillus* sp. | 25000,00 |  |  |
|  | C | VCZ | *Mucor* sp. | 9,25 | 0,00 | 1,00 |  |  | Total | | | | 62500,00 |  |  |
|  | Total | | | 9,25 |  |  |  |  | CA | | DG18 37ºC | *Mucor* sp. | 1000,00 | 1,09 | 2,62 |
|  | M | VCZ | *Mucor* sp. | 10,48 | 0,00 | 1,00 |  |  | CA | | DG18 37ºC | *Paecilomyces* sp. | 6000,00 |  |  |
|  | Total | | | 10,48 |  |  |  |  | CA | | DG18 37ºC | *Penicillium* sp. | 1000,00 |  |  |
|  | P | VCZ | *Mucor* sp. | 9,83 | 0,00 | 1,00 |  |  | CA | | DG18 37ºC | *Aspergillus* sp. | 6500,00 |  |  |
|  | Total | | | 9,83 |  |  |  |  | Total | | | | 14500,00 |  |  |

| Matrice | Sampling Place | Culture media | | Genera/Species | | Culture Media (CFU/m-2/-3) | | Shannon Index (H) | Simpson Index (D) | |  | | Matrice | Sampling Place | Culture media | Genera/Species | Culture Media (CFU/m-2/-3) | Shannon Index (H) | Simpson Index (D) |
| --- | --- | --- | --- | --- | --- | --- | --- | --- | --- | --- | --- | --- | --- | --- | --- | --- | --- | --- | --- |
|  |  |  |  |  |  |  |  |  |  |  |  | |  |  |  |  |  |  |  |
| Filter | CD | DG18 37ºC | | *Paecilomyces* sp. | | 3500,00 | | 1,09 | 2,68 | |  | | Swab | CA | MEA | *Cladosporium* sp. | 60000,00 | 1,53 | 4,28 |
|  | CD | DG18 37ºC | | *Penicillium* sp. | | 8500,00 | |  |  | |  | |  | CA | MEA | *Fusarium solani* | 160000,00 |  |  |
|  | CD | DG18 37ºC | | *Rhizopus* sp. | | 500,00 | |  |  | |  | |  | CA | MEA | *Penicillium* sp. | 130000,00 |  |  |
|  | CD | DG18 37ºC | | *Aspergillus* sp. | | 11000,00 | |  |  | |  | |  | CA | MEA | *Rhizopus* sp. | 60000,00 |  |  |
|  | Total | | | | | 23500,00 | |  |  | |  | |  | CA | MEA | *Trichoderma* sp. | 80000,00 |  |  |
|  | M | DG18 37ºC | | *C.sitophila* | | 2000,00 | | 0,91 | 2,18 | |  | |  | Total | | | 490000,00 |  |  |
|  | M | DG18 37ºC | | *Mucor* sp. | | 42500,00 | |  |  | |  | |  | CD | MEA | *Aureobasium* sp. | 10000,00 | 0,87 | 1,92 |
|  | M | DG18 37ºC | | *Paecilomyces* sp. | | 6000,00 | |  |  | |  | |  | CD | MEA | *Cladosporium* sp. | 90000,00 |  |  |
|  | M | DG18 37ºC | | *Aspergillus* sp. | | 66500,00 | |  |  | |  | |  | CD | MEA | *Penicillium* sp. | 160000,00 |  |  |
|  | Total | | | | | 117000,00 | |  |  | |  | |  | CD | MEA | *Trichoderma* sp. | 570000,00 |  |  |
|  | P | DG18 37ºC | | *Mucor* sp. | | 500,00 | | 0,83 | 2,03 | |  | |  | Total | | | 830000,00 |  |  |
|  | P | DG18 37ºC | | *Penicillium* sp. | | 2500,00 | |  |  | |  | |  | M | MEA | *Geomyces* sp. | 70000,00 | 0,99 | 2,37 |
|  | P | DG18 37ºC | | *Aspergillus* sp. | | 5000,00 | |  |  | |  | |  | M | MEA | *Penicillium* sp. | 710000,00 |  |  |
|  | Total | | | | | 8000,00 | |  |  | |  | |  | M | MEA | *Rhizopus* sp. | 60000,00 |  |  |
|  | CA | SDA | | *Chrysosporium* sp. | | 500,00 | | 0,60 | 1,62 | |  | |  | M | MEA | *Trichoderma* sp. | 660000,00 |  |  |
|  | CA | SDA | | *Penicillium sp.* | | 41500,00 | |  |  | |  | |  | Total | | | 1500000,00 |  |  |
|  | CA | SDA | | *Rhizopus* sp. | | 13500,00 | |  |  | |  | |  | P | MEA | *Cladosporium* sp. | 40000,00 | 0,89 | 1,86 |
|  | Total | | | | | 55500,00 | |  |  | |  | |  | P | MEA | *Penicillium* sp. | 120000,00 |  |  |
|  | CD | SDA | | *Cladosporium* sp. | | 500,00 | | 0,19 | 1,10 | |  | |  | P | MEA | *Rhizopu*s sp. | 60000,00 |  |  |
|  | CD | SDA | | *Penicillium* sp. | | 10000,00 | |  |  | |  | |  | P | MEA | *Aspergillus* sp. | 540000,00 |  |  |
|  | Total | | | | | 10500,00 | |  |  | |  | |  | Total | | | 760000,00 |  |  |
|  | M | SDA | | *Aspergillus* sp. | | 1000,00 | | 0,84 | 1,85 | |  | |  | CA | DG18 | *Cladosporium* sp. | 10000,00 | 0,67 | 1,65 |
|  | M | SDA | | *C.sitophila* | | 37000,00 | |  |  | |  | |  | CA | DG18 | *Penicillium* sp. | 180000,00 |  |  |
|  | M | SDA | | *Mucor* sp. | | 1000,00 | |  |  | |  | |  | CA | DG18 | *Aspergillus* sp. | 50000,00 |  |  |
|  | M | SDA | | *Penicillium* sp. | | 159500,00 | |  |  | |  | |  | Total | | | 240000,00 |  |  |
|  | M | SDA | | *Rhizopus* sp. | | 27000,00 | |  |  | |  | |  | CD | DG18 | *Chrysopsorium* sp. | 80000,00 | 1,13 | 2,54 |
|  | Total | | | | | 225500,00 | |  |  | |  | |  | CD | DG18 | *Cladosporium* sp. | 180000,00 |  |  |
|  | P | SDA | | *Penicillium* sp. | | 11500,00 | | 0,00 | 1,00 | |  | |  | CD | DG18 | *Penicillium* sp. | 600000,00 |  |  |
|  | Total | | | | | 11500,00 | |  |  | |  | |  | CD | DG18 | *Aspergillus* sp. | 190000,00 |  |  |
|  | M | ITZ | | *C.sitophila* | | 1000,00 | | 0,00 | 1,00 | |  | |  | Total | | | 1050000,00 |  |  |
|  | Total | | | | | 1000,00 | |  |  | |  | |  | M | DG18 | *C.sitophila* | 60000,00 | 0,90 | 1,87 |
|  | CD | VCZ | | *Mucor* sp. | | 1000,00 | | 0,00 | 1,00 | |  | |  | M | DG18 | *Cladosporium* sp. | 50000,00 |  |  |
|  | Total | | | | | 1000,00 | |  |  | |  | |  | M | DG18 | *Penicillium* sp. | 560000,00 |  |  |
|  | M | VCZ | | *C.sitophila* | | 18500,00 | | 0,20 | 1,11 | |  | |  | M | DG18 | *Aspergillus* sp. | 120000,00 |  |  |
|  | M | VCZ | | *Mucor* sp. | | 1000,00 | |  |  | |  | |  | Total | | | 790000,00 |  |  |
|  | Total | | | | | 19500,00 | |  |  | |  | |  | P | DG18 | *C.sitophila* | 30000,00 | 0,84 | 1,92 |
|  | P | VCZ | | *Mucor* sp. | | 500,00 | | 0,00 | 1,00 | |  | |  | P | DG18 | *Cladosporium* sp. | 30000,00 |  |  |
|  | Total | | | | | 500,00 | |  |  | |  | |  | P | DG18 | *Penicillium* sp. | 240000,00 |  |  |
|  | M | PSZ | | *Mucor* sp. | | 500,00 | | 0,00 | 1,00 | |  | |  | P | DG18 | *Aspergillus* sp. | 610000,00 |  |  |
|  | Total | | | | | 500,00 | |  |  | |  | |  | Total | | | 910000,00 |  |  |
| Gloves | C | MEA | | *Fusarium solani* | | 500,00 | | 0,83 | 1,74 | |  | |  | CA | DG18 37ºC | *C.sitophila* | 10000,00 | 1,03 | 2,25 |
|  | C | MEA | | *Mucor* sp. | | 500,00 | |  |  | |  | |  | CA | DG18 37ºC | *Paecilomyces* sp. | 10000,00 |  |  |
|  | C | MEA | | *Rhizopus* sp. | | 7000,00 | |  |  | |  | |  | CA | DG18 37ºC | *Penicillium* sp. | 30000,00 |  |  |
|  | C | MEA | | *Aspergillus* sp. | | 1500,00 | |  |  | |  | |  | CA | DG18 37ºC | *Aspergillus* sp. | 80000,00 |  |  |
|  | Total | | | | | 9500,00 | |  |  | |  | |  | Total | | | 130000,00 |  |  |
|  | C | DG18 | | *Penicillium* sp. | | 23500,00 | | 0,41 | 1,33 | |  | |  | CD | DG18 37ºC | *Mucor* sp. | 70000,00 | 1,03 | 2,05 |
|  | C | DG18 | | *Aspergillus* sp. | | 4000,00 | |  |  | |  | |  | CD | DG18 37ºC | *Paecilomyces* sp. | 20000,00 |  |  |
|  | Total | | | | | 27500,00 | |  |  | |  | |  | CD | DG18 37ºC | *Penicillium* sp. | 20000,00 |  |  |
|  | C | DG18 37ºC | | *Mucor* sp. | | 500,00 | | 0,35 | 1,19 | |  | |  | CD | DG18 37ºC | *Trichoderma* sp. | 70000,00 |  |  |
|  | C | DG18 37ºC | | *Paecilomyces* sp. | | 1000,00 | |  |  | |  | |  | CD | DG18 37ºC | *Aspergillus* sp. | 370000,00 |  |  |
|  | C | DG18 37ºC | | *Aspergillus* sp. | | 16000,00 | |  |  | |  | |  | Total | | | 550000,00 |  |  |
|  | Total | | | | | 17500,00 | |  |  | |  | |  | M | DG18 37ºC | *Paecilomyces* sp. | 40000,00 | 0,27 | 1,14 |
| Masks | C | DG18 | | *Penicillium* sp. | | 1000,00 | | 0,53 | 1,53 | |  | |  | M | DG18 37ºC | *Trichoderma* sp. | 10000,00 |  |  |
|  | C | DG18 | | *Aspergillus* sp. | | 3500,00 | |  |  | |  | |  | M | DG18 37ºC | *Aspergillus* sp. | 720000,00 |  |  |
|  | Total | | | | | 4500,00 | |  |  | |  | |  | Total | | | 770000,00 |  |  |
|  | CL | DG18 | | *Penicillium* sp. | | 2500,00 | | 0,00 | 1,00 | |  | |  | P | DG18 37ºC | *Paecilomyces* sp. | 20000,00 | 0,22 | 1,12 |
|  | Total | | | | | 2500,00 | |  |  | |  | |  | P | DG18 37ºC | *Aspergillus* sp. | 330000,00 |  |  |
|  | C | DG18 37ºC | | *Aspergillus* sp. | | 1000,00 | | 0,00 | 1,00 | |  | |  | Total | | | 350000,00 |  |  |
|  | Total | | | | | 1000,00 | |  |  | |  | | Andersen | CA | DG18 | *Aspergillus* sp. | 714,57 | 0,82 | 1,74 |
|  | CL | DG18 37ºC | | *C.sitophila* | | 500,00 | | 0,00 | 1,00 | |  | |  | CA | DG18 | *C.sitophila* | 141,34 |  |  |
|  | Total | | | | | 500,00 | |  |  | |  | |  | CA | DG18 | *Cladosporium* sp. | 789,16 |  |  |
| SD | C | MEA | | *C.sitophila* | | 42,00 | | 1,15 | 2,77 | |  | |  | CA | DG18 | *Penicillium* sp. | 4648,61 |  |  |
|  | C | MEA | | *Penicillium* sp. | | 143,00 | |  |  | |  | |  | Total | | | 6293,68 |  |  |
|  | C | MEA | | *Trichoderma* sp. | | 192,00 | |  |  | |  | |  | CA | DG18 37ºC | *Aspergillus* sp. | 1009,03 | 0,91 | 1,87 |
|  | C | MEA | | *Aspergillus* sp. | | 31,00 | |  |  | |  | |  | CA | DG18 37ºC | *C.sitophila* | 157,05 |  |  |
|  | Total | | | | | 408,00 | |  |  | |  | |  | CA | DG18 37ºC | *Mucor* sp. | 176,68 |  |  |
|  | P | MEA | | *C.sitophila* | | 4,00 | | 0,70 | 1,61 | |  | |  | CA | DG18 37ºC | *Paecilomyces* sp. | 78,52 |  |  |
|  | P | MEA | | *Cladosporium* sp. | | 4,00 | |  |  | |  | |  | Total | | | 1421,28 |  |  |
|  | P | MEA | | *Trichoderma* sp. | | 27,00 | |  |  | |  | |  | CD | DG18 | *Aspergillus* sp. | 1173,93 | 0,88 | 1,91 |
|  | Total | | | | | 35,00 | |  |  | |  | |  | CD | DG18 | *Cladosporium* sp. | 922,65 |  |  |
|  | C | DG18 | | *Cladosporium* sp. | | 116,00 | | 1,22 | 3,04 | |  | |  | CD | DG18 | *Paecilomyces* sp. | 141,34 |  |  |
|  | C | DG18 | | *Paecilomyces* sp. | | 43,00 | |  |  | |  | |  | CD | DG18 | *Penicillium* sp. | 5076,56 |  |  |
|  | C | DG18 | | *Penicillium* sp. | | 280,00 | |  |  | |  | |  | Total | | | 7314,49 |  |  |
|  | C | DG18 | | *Aspergillus* sp. | | 182,00 | |  |  | |  | |  | CD | DG18 37ºC | *Aspergillus* sp. | 1016,88 | 1,13 | 2,54 |
|  | Total | | | | | 621,00 | |  |  | |  | |  | CD | DG18 37ºC | *Mucor* sp. | 314,10 |  |  |
|  | P | DG18 | | *Cladosporium* sp. | | 3,00 | | 0,70 | 1,63 | |  | |  | CD | DG18 37ºC | *Paecilomyces* sp. | 137,42 |  |  |
|  | P | DG18 | | *Penicillium* sp. | | 26,00 | |  |  | |  | |  | CD | DG18 37ºC | *Penicillium* sp. | 310,17 |  |  |
|  | P | DG18 | | Aspergillus sp. | | 5,00 | |  |  | |  | |  | Total | | | 1778,56 |  |  |
|  | Total | | | | | 34,00 | |  |  | |  | |  | M | DG18 | *Aspergillus* sp. | 1103,26 | 0,52 | 1,38 |
|  | C | DG18 37ºC | | *Mucor* sp. | | 6,00 | | 0,41 | 1,22 | |  | |  | M | DG18 | *C.sitophila* | 94,23 |  |  |
|  | C | DG18 37ºC | | *Paecilomyces* sp. | | 12,00 | |  |  | |  | |  | M | DG18 | *Chrysosporium* sp. | 58,89 |  |  |
|  | C | DG18 37ºC | | *Rhizopus* sp. | | 4,00 | |  |  | |  | |  | M | DG18 | *Penicillium* sp. | 6489,99 |  |  |
|  | C | DG18 37ºC | | *Aspergillus* sp. | | 207,00 | |  |  | |  | |  | Total | | | 7746,37 |  |  |
|  | Total | | | | | 229,00 | |  |  | |  | |  | M | DG18 37ºC | *Aspergillus* sp. | 2316,45 | 0,67 | 1,48 |
|  | P | DG18 37ºC | | *Aspergillus* sp. | | 24,00 | | 0,00 | 1,00 | |  | |  | M | DG18 37ºC | *Mucor* sp. | 282,69 |  |  |
|  | Total | | | | | 24,00 | |  |  | |  | |  | M | DG18 37ºC | *Paecilomyces* sp. | 109,93 |  |  |
|  | C | SDA | | *Aspergillus* sp. | | 15,00 | | 1,01 | 2,55 | |  | |  | M | DG18 37ºC | *Penicillium* sp. | 137,42 |  |  |
|  | C | SDA | | *Mucor* sp. | | 7,00 | |  |  | |  | |  | Total | | | 2846,49 |  |  |
|  | C | SDA | | *Rhizopus* sp. | | 22,00 | |  |  | |  | | MAS100 | CA | MEA | *Cladosporium* sp. | 395,00 | 0,90 | 1,96 |
|  | Total | | | | | 44,00 | |  |  | |  | |  | CA | MEA | *Paecilomyces* sp. | 530,00 |  |  |
|  | C | ITZ | | *Mucor* sp. | | 8,00 | | 0,50 | 1,47 | |  | |  | CA | MEA | *Penicillium* sp. | 2090,00 |  |  |
|  | C | ITZ | | *Rhizopus* sp. | | 2,00 | |  |  | |  | |  | CA | MEA | *Trichoderma* sp. | 55,00 |  |  |
|  | Total | | | | | 10,00 | |  |  | |  | |  | Total | | | 3070,00 |  |  |
|  | C | VCZ | | *Mucor* sp. | | 5,00 | | 0,64 | 1,80 | |  | |  | CA | DG18 | *Cladosporium* sp. | 1180,00 | 0,69 | 1,69 |
|  | C | VCZ | | *Rhizopus* sp. | | 10,00 | |  |  | |  | |  | CA | DG18 | *Monascus ruber* | 75,00 |  |  |
|  | Total | | | | | 15,00 | |  |  | |  | |  | CA | DG18 | *Penicillium* sp. | 3575,00 |  |  |
|  | C | PSZ | | *Mucor* sp. | | 14,00 | | 0,53 | 1,53 | |  | |  | CA | DG18 | *Aspergillus* sp. | 65,00 |  |  |
|  | C | PSZ | | *Rhizopus* sp. | | 4,00 | |  |  | |  | |  | Total | | | 4895,00 |  |  |
|  | Total | | | | | 18,00 | |  |  | |  | |  |  |  |  |  |  |  |
| MAS100 | CD | MEA | *Cladosporium* sp. | | 185,00 | | 1,04 | | | 2,49 | |  |  |  |  |  |  |  |  |
|  | CD | MEA | *Paecilomyces* sp. | | 75,00 | |  | | |  | |  |  |  |  |  |  |  |  |
|  | CD | MEA | *Penicillium* sp. | | 1075,00 | |  | | |  | |  |  |  |  |  |  |  |  |
|  | CD | MEA | *Trichoderma* sp. | | 855,00 | |  | | |  | |  |  |  |  |  |  |  |  |
|  | Total | | | | 2190,00 | |  | | |  | |  |  |  |  |  |  |  |  |
|  | CD | DG18 | *C.sitophila* | | 225,00 | | 1,03 | | | 2,29 | |  |  |  |  |  |  |  |  |
|  | CD | DG18 | *Cladosporium* sp. | | 1235,00 | |  | | |  | |  |  |  |  |  |  |  |  |
|  | CD | DG18 | *Penicillium* sp. | | 3540,00 | |  | | |  | |  |  |  |  |  |  |  |  |
|  | CD | DG18 | *Aspergillus* sp. | | 825,00 | |  | | |  | |  |  |  |  |  |  |  |  |
|  | Total | | | | 5825,00 | |  | | |  | |  |  |  |  |  |  |  |  |
|  | M | MEA | *Cladosporium* sp. | | 470,00 | | 0,99 | | | 2,05 | |  |  |  |  |  |  |  |  |
|  | M | MEA | *Penicillium* sp. | | 2220,00 | |  | | |  | |  |  |  |  |  |  |  |  |
|  | M | MEA | *Rhizopus* sp. | | 355,00 | |  | | |  | |  |  |  |  |  |  |  |  |
|  | M | MEA | *Trichoderma* sp. | | 265,00 | |  | | |  | |  |  |  |  |  |  |  |  |
|  | Total | | | | 3310,00 | |  | | |  | |  |  |  |  |  |  |  |  |
|  | M | DG18 | *Cladosporium* sp. | | 1790,00 | | 0,58 | | | 1,47 | |  |  |  |  |  |  |  |  |
|  | M | DG18 | *Penicillium* sp. | | 8800,00 | |  | | |  | |  |  |  |  |  |  |  |  |
|  | M | DG18 | *Trichoderma* sp. | | 170,00 | |  | | |  | |  |  |  |  |  |  |  |  |
|  | M | DG18 | *Aspergillus* sp. | | 115,00 | |  | | |  | |  |  |  |  |  |  |  |  |
|  | Total | | | | 10875,00 | |  | | |  | |  |  |  |  |  |  |  |  |
|  | P | MEA | *Aureobasidium* sp. | | 140,00 | | 0,84 | | |  | |  |  |  |  |  |  |  |  |
|  | P | MEA | *Cladosporium* sp. | | 690,00 | |  | | | 1,80 | |  |  |  |  |  |  |  |  |
|  | P | MEA | *Penicillium* sp. | | 2740,00 | |  | | |  | |  |  |  |  |  |  |  |  |
|  | P | MEA | *Trichoderma* sp. | | 245,00 | |  | | |  | |  |  |  |  |  |  |  |  |
|  | Total | | | | 3815,00 | |  | | |  | |  |  |  |  |  |  |  |  |
|  | P | DG18 | *Cladosporium* sp. | | 720,00 | | 1,13 | | | 2,60 | |  |  |  |  |  |  |  |  |
|  | P | DG18 | *Penicillium* sp. | | 1930,00 | |  | | |  | |  |  |  |  |  |  |  |  |
|  | P | DG18 | *Trichoderma* sp. | | 200,00 | |  | | |  | |  |  |  |  |  |  |  |  |
|  | P | DG18 | *Aspergillus* sp. | | 650,00 | |  | | |  | |  |  |  |  |  |  |  |  |
|  | Total | | | | 3500,00 | |  | | |  | |  |  |  |  |  |  |  |  |
|  | E | MEA | *Aureobasidium* sp. | | 45,00 | | 0,92 | | | 2,22 | |  |  |  |  |  |  |  |  |
|  | E | MEA | *Chrysosporium* sp. | | 60,00 | |  | | |  | |  |  |  |  |  |  |  |  |
|  | E | MEA | *Cladosporium* sp. | | 1330,00 | |  | | |  | |  |  |  |  |  |  |  |  |
|  | E | MEA | *Penicillium* sp. | | 1355,00 | |  | | |  | |  |  |  |  |  |  |  |  |
|  | E | MEA | *Aspergillus* sp. | | 45,00 | |  | | |  | |  |  |  |  |  |  |  |  |
|  | Total | | | | 2835,00 | |  | | |  | |  |  |  |  |  |  |  |  |
|  | E | DG18 | *C.sitophila* | | 90,00 | | 0,99 | | | 2,30 | |  |  |  |  |  |  |  |  |
|  | E | DG18 | *Cladosporium* sp. | | 1025,00 | |  | | |  | |  |  |  |  |  |  |  |  |
|  | E | DG18 | *Penicillium* sp. | | 630,00 | |  | | |  | |  |  |  |  |  |  |  |  |
|  | E | DG18 | *Aspergillus* sp. | | 90,00 | |  | | |  | |  |  |  |  |  |  |  |  |
|  | Total | | | | 1835,00 | |  | | |  | |  |  |  |  |  |  |  |  |
